# Review and Evaluation of the Potential Health Effects of Oxidic Nickel Nanoparticles

**DOI:** 10.3390/nano11030642

**Published:** 2021-03-05

**Authors:** Sharlee L. More, Michael Kovochich, Tara Lyons-Darden, Michael Taylor, Alexandra M. Schulte, Amy K. Madl

**Affiliations:** 1 Cardno ChemRisk, 6720 S Macadam Ave Suite 150, Portland, OR 97219, USA; 2 Cardno ChemRisk, 30 North LaSalle St Suite 3910, Chicago, IL 60602, USA; michael.kovochich@cardno.com; 3 NiPERA, 2525 Meridian Parkway, Suite 240, Durham, NC 27713, USA; TLyons-Darden@nipera.org (T.L.-D.); mtaylor@nipera.org (M.T.); 4 Cardno ChemRisk, 65 Enterprise Drive Suite 150, Aliso Viejo, CA 92656, USA; alex.schulte@cardno.com (A.M.S.); amy.madl@cardno.com (A.K.M.)

**Keywords:** nickel oxide nanoparticles, oxidic nickel nanoparticles, toxicity, nanotoxicity

## Abstract

The exceptional physical and chemical properties of nickel nanomaterials have been exploited in a range of applications such as electrical conductors, batteries, and biomaterials. However, it has been suggested that these unique properties may allow for increased bioavailability, bio-reactivity, and potential adverse health effects. Thus, the purpose of this review was to critically evaluate data regarding the toxicity of oxidic nickel nanoparticles (nickel oxide (NiO) and nickel hydroxide (Ni(OH)_2_) nanoparticles) with respect to: (1) physico-chemistry properties; (2) nanomaterial characterization in the defined delivery media; (3) appropriateness of model system and translation to potential human effects; (4) biodistribution, retention, and clearance; (5) routes and relevance of exposure; and (6) current research data gaps and likely directions of future research. Inhalation studies were prioritized for review as this represents a potential exposure route in humans. Oxidic nickel particle size ranged from 5 to 100 nm in the 60 studies that were identified. Inflammatory responses induced by exposure of oxidic nickel nanoparticles via inhalation in rodent studies was characterized as acute in nature and only displayed chronic effects after relatively large (high concentration and long duration) exposures. Furthermore, there is no evidence, thus far, to suggest that the effects induced by oxidic nickel nanoparticles are related to preneoplastic events. There are some data to suggest that nano- and micron-sized NiO particles follow a similar dose response when normalized to surface area. However, future experiments need to be conducted to better characterize the exposure–dose–response relationship according to specific surface area and reactivity as a dose metric, which drives particle dissolution and potential biological responses.

## 1. Introduction

Nanoparticles are generally defined as particles with one or more of three dimensions measuring below 100 nanometers (nm). Due to their small size, nanoparticles have a large surface area per unit of mass and the potential for greater particle surface-cell interactions than particles of micron size. Nanomaterials, including nickel-containing nanomaterials, have a variety of unique characteristics, including high surface area per mass, low boiling point, low melting point, high surface energy, and magnetism. The exceptional physical and chemical properties of nickel nanomaterials have been exploited in a range of applications including electrical conductors, magnetic materials, fuel cells, pigmentation, alloys, batteries, catalytic reactions, and biomedical materials [1]. It has been suggested that the unique properties of nickel nanomaterials may allow for increased bioavailability, bio-reactivity, and potential adverse health effects. Therefore, understanding the material characteristics that play a role in biological responses will help understand the potential health effects associated with nickel-containing nanomaterials.

While inhalation and dermal contact are the primary routes of airborne nickel exposure in occupational or industrial settings, the use of nickel in biomedical materials may provide opportunities for exposure scenarios that involve direct contact with tissue via implantation [2]. The general public is not typically exposed to nickel nanoparticles in the airborne state due to their primary use in specialized applications and end products (e.g., lithium ion batteries, conductive coatings, pigments, additives, fuel cells, electrodes, etc.) in which they are generally tightly bound within a matrix and not likely to be released into the air or on product surfaces [3,4]. This greatly reduces the potential for inhalation or dermal exposure by the public (consumers). However, occupational exposure is possible in manufacturing and research facilities during production and handling of nickel nanoparticles, where aerosols of nickel nanoparticles can be generated.

The toxicity associated with nickel compounds, including nickel oxide (NiO), has been widely studied [5,6,7]. Respiratory toxicity and cancer, skin sensitization, and reproductive effects have been reported in human and/or animal studies exposed to some nickel compounds. The bioavailability of the Ni^2+^ ion at target sites is considered to play a role in the differences observed between human toxicity potencies/potentials of different nickel chemical forms [8]. Thus, the purpose of this manuscript is to identify and critically evaluate relevant literature with respect to the health effects of NiO and nickel hydroxide (Ni(OH)_2_) nanomaterials (referred to as oxidic nickel). Further, the evaluation aimed to understand whether the toxicity of oxidic nickel nanomaterials may be similar to micron-sized oxidic nickel particle toxicity when normalized to surface area or whether unique characteristics due to the nanometer size influence the distinctive biological responses. Both in vitro and in vivo mammalian toxicity studies, in particular inhalation and instillation studies, have aimed to evaluate the key health endpoints for oxidic nickel nanomaterials. These studies have focused on several health effects including pulmonary inflammation, oxidative stress, cytotoxicity, genotoxicity, systemic effects, and cardiovascular effects. Physical and chemical characteristics of oxidic nickel nanomaterials, including chemical form, dissolution rate, particle size, cellular uptake, protein adsorption, surface morphology, and production method likely play a role to some degree in the above-mentioned health effects. Therefore, this analysis will describe the current state-of-the-science of oxidic nickel nanomaterials and their effects in in vitro and in vivo mammalian toxicity studies with respect to: (1) physico-chemistry (e.g., surface chemistry, dissolution, size distribution, morphological characteristics); (2) quality of nanomaterial characterization in the defined delivery media; (3) appropriateness of model system and extrapolation to potential human effects; (4) biodistribution, retention, and clearance; (5) routes of exposure and target organ effects; and (6) current research data gaps and recommended directions of future research.

## 2. Literature Review

**Figure 1:**
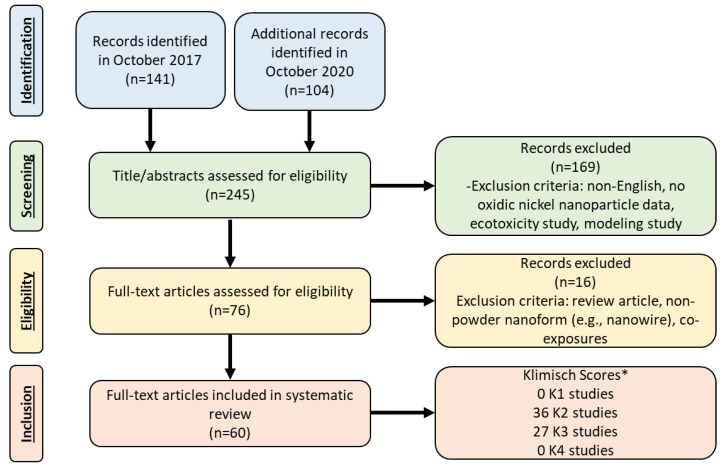
Flow chart of literature review and study selection following inclusion and exclusion criteria. Articles that were identified by the process were included for the full-article review. Replicate entries were removed. * Klimisch scores were evaluated twice for studies with both in vivo and in vitro data.

A standardized protocol was followed to perform a systematic literature review to identify in vitro and in vivo mammalian toxicity studies associated with NiO and Ni(OH)_2_ nanoparticles. The following scientific databases were used to search for publications relevant to the potential toxicity of oxidic nickel nanomaterials: PUBMED, TOXLINE, SCISEARCH, MEDLINE, NTIS, Google Scholar, and the International Council on Nanotechnology Virtual Journal of Nanotechnology Environment, Health and Safety online database. The key words used for search terms included: nickel oxide nanoparticle, nickel oxide nanomaterial, nickel hydroxide nanoparticle, nickel hydroxide nanomaterial, oxidic nickel nanoparticles, oxidic nickel nanomaterials, toxicity, biokinetics, dissolution, and solubility. Inclusion criteria was applied to identify relevant in vitro and in vivo studies involving: evaluated nickel oxide nanoparticles and nickel hydroxide nanoparticles; utilized mammalian test systems; reported original research; and published in English. Studies that examined nanowires were excluded from this analysis. A literature search was conducted through October 2017. From the 141 articles that were returned, 48 were included in this analysis (Figure 1). An additional literature search was conducted through October 2020 to identify pertinent manuscripts that had been published since the original literature search and examined outcomes related to in vivo exposure and/or genotoxicity (including in vitro genotoxicity studies). Of the 104 articles that were found in the October 2020 literature search, twelve papers were identified and included. A cursory review was performed with the excluded in vitro studies published from 2017 to 2020. Based on a review and comparison with the studies published prior to 2017, none of the themes or conclusions changed based on the more recent literature.

A total of 60 articles were identified. A comprehensive reference table for the identified studies was developed to describe the in vitro and in vivo data regarding the nanoparticle characterization (Table 1 and Supplemental Table S1), experimental design, and assay/health endpoint (Tables 2–4). The articles obtained from the literature search were categorized based on in vitro (Supplemental Table S2) and in vivo (Tables 2–4) studies. The papers were made up of 17 in vitro studies and 43 in vivo studies, including three studies which utilized both in vitro and in vivo systems. No studies were identified that investigated the reproductive and developmental toxicity of oxidic nickel nanoparticles to this date.

## 3. Study Quality Assessment

The studies were objectively evaluated and ranked according to the methods developed by Klimisch et al. [9], Card and Magnuson [10] and Sayes and Warheit [11]. In brief, according to Klimisch et al. [9], the criteria for evaluating study quality include the following factors: (1) test substance identification, (2) test organism characterization (3) study design, (4) documentation of study results, and (5) reliability and plausibility (relevance) of the study. Furthermore, as described by Card and Magnuson [10] and Sayes and Warheit [11], key nanomaterial characteristics such as particle size, size distribution, agglomeration, morphology, composition, crystallinity, purity, surface area, charge, surface chemistry, and characterization in relevant media were noted when available (though not all required for inclusion in this manuscript). The physicochemical nanoparticle characteristics examined in the various studies is provided in Supplemental Table S1. Specifically, nanomaterials characterization was considered in the test substance identification and studies that did not perform thorough nanoparticle characterization (including nanoparticle size and characterization in relevant media) were scored lower in this criteria. The papers that presented both in vivo and in vitro data were ranked twice; once for the in vivo data and once for the in vitro data. It should be noted, that while Klimisch scores were used to evaluate the study quality, studies were not excluded from discussion in this manuscript based on the Klimisch scores.

The methods developed by Klimisch et al. [9], Card and Magnuson [10] and Sayes and Warheit [11] essentially utilize a ranking system to categorize the study score based on a series of objective criteria and produces a K-score of K1, K2, K3 or K4. A score of K1 represents studies that are reliable without restrictions, K2 represents studies that are reliable with restrictions, K3 represents studies that are unreliable, and K4 represents studies in which there is insufficient information presented with which to assess reliability. Zero studies were scored as K1 (reliable without restrictions). Eighteen in vivo studies and 18 in vitro studies were ranked as K2. Two in vitro and 25 in vivo studies were ranked as K3. Zero K4 studies were identified since K4 studies were excluded during the literature search based on the inclusion/exclusion criteria. In addition, 29 studies investigated the toxicity associated with one dose and hence were ranked as a K3 due to lack of dose–response information. The in vitro studies more frequently involved less detailed or complex protocols for assays, and also were less likely to conduct detailed nanoparticle characterization. In addition, some of the in vitro and in vivo studies which scored as K3 failed to adequately characterize the nanoparticles in a way that would allow for meaningful interpretations of the biological data.

## 4. Nanomaterial Physicochemical Characteristics

Physical and chemical characteristics of oxidic nickel nanomaterials, including chemical form, ion dissolution, and particle size may play a role in potential biological responses observed in the various studies described below. Therefore, the toxicological endpoints were considered in relation to physico-chemistry properties, such as surface chemistry, agglomeration, and dissolution. The scope of our review did not include an analysis of different nanoparticle synthesis and engineering techniques, including the use of any capping agents.

Table 1 outlines the nanoparticle primary particle size and size in media used in the toxicity studies. Supplemental Table S1 provides a list of the other nanoparticle characteristics investigated in the studies. Overall, the NiO and Ni(OH)_2_ particle size ranged from 5 to 100 nm. Particle sizes in relevant media ranged from 20 nm to 8.69 µm in in vivo studies. Size in relevant media ranged from 38 to 750 nm in in vitro studies. It is known that nanoparticle materials are likely to agglomerate and this process can affect the various biological responses both in vitro and in vivo. Agglomerated nanoparticles will behave differently from well-dispersed particles due to differences in effective particle size, shape, and ultimately biological reactivity. Surface chemistry, including the adsorption of proteins can affect the agglomeration state of nanoparticles and influence biological responses. As such, the treatment of nanoparticles prior to a toxicological study can affect the reported outcome. Each research lab performed slightly different dispersion techniques when examining the biological response to oxidic nickel nanoparticles under in vitro or in vivo testing conditions. Many of the studies used dispersion media to produce well-dispersed nanoparticles for instillation and pharyngeal aspiration experiments in vivo [12,13,14,15]. For example, Sager et al. [15] dispersed NiO nanoparticles in four different media: phosphate buffered saline; dispersion media that mimicked diluted alveolar lining fluid; and two different surfactants. Well-dispersed NiO nanoparticles were found to induce a greater inflammatory effect than agglomerated nanoparticles when instilled or aspirated into rats [12,15]. Unfortunately, further analysis on factors such as ion release of agglomerated versus well-dispersed particles was not investigated. Agglomeration may also affect the dose rate in in vitro settings due to increased sedimentation rates [16], but, this parameter was not explored in the majority of current literature. Gutierrez et al. [17] compared the effects of in vitro dispersion techniques on the nanoparticles physicochemistry and toxicological effects and found variability in gene expression of oxidative stress markers (including hemoxygenase 1) after exposing lung cell lines (A549 and 16HBE14o) to 0.01 to 100 µg/mL NiO nanoparticles (<20 nm). They reported that the dispersion media differentially affected nanoparticle physicochemical properties and toxicity and that dispersion media with Fetal Bovine Serum (FBS) kept the nanoparticles better dispersed as compared to media with surfactants.

Dissolution of metal particles is an important parameter of metal toxicology and likely plays a role in biological responses to oxidic nickel nanoparticles [18]. Horie et al. [19] observed a greater release of nickel ions in nanoparticles compared with larger sized particles based on equivalent mass doses. Furthermore, cytotoxicity was also correlated with the extent of ion release from both nano- versus micron-sized particles as well as black versus green crystalline forms of NiO nanoparticles. Shinohara et al. [20] reported irregular shaped NiO nanoparticles (140 nm) dissolved less than spherical NiO nanoparticles (20 nm). Cho et al. [12], Latvala et al. [21], and Shinohara et al. [20] observed a greater release of nickel ions from NiO nanoparticles under acidic circumstances compared with neutral or basic conditions. Additionally, Gillespie et al. [22] determined the dissolution of Ni(OH)_2_ nanoparticles by measuring the dissolution rate of particles in a flow-through dialysis systems. The authors reported a dissolution rate of 85% and 95% for Ni(OH)_2_ nanoparticles after 24 h in lung and lysosomal fluid, respectively. Nishi et al. [23] reported that NiO nanoparticles (26 nm) dissolved in artificial lysosomal fluid in approximately one week. In contrast, they reported that NiO nanoparticles were observed in cells in rat lungs up to 1 month; the authors thus concluded that dissolution took over one month in in vivo systems. As discussed above, the particle size and surface area differences may have contributed to these effects, particularly when equivalent mass doses were delivered. Other factors besides surface area will influence dissolution of oxidic nickel nanoparticles including the chemistry of the surrounding media. Factors such as protein content and pH affect dissolution rates of metal nanoparticles.

**Table 1:** Primary particle size and size in media of nanoparticles used in the studies investigating the toxicity of oxidic nickel nanoparticles.

Study	Nanoparticle Type	Oxidic Nickel Nanoparticle Primary Particle Size (Average ± SD)	Oxidic Nickel Nanoparticle Size in Media (Average ± SD)
Abudayyak et al. [24]	NiO	15.0 ± 7.54 nm	135.81 nm
Ada et al. [25]	NiO	20 nm	NR
Åkerlund et al. [26]	NiO	<50 nm	200 nm
Ali [27]	NiO	<50 nm	91.54 nm
Bai et al. [28]	NiO	20 nm	685.7 nm
Cao et al. [29]	NiO	18.6 ± 5.5 nm	313 ± 12.6 nm
Capasso et al. [30]	NiO	50 nm	80 and 450 nm
Cho et al. [13]	NiO	10–20 nm	92 nm
Cho et al. [12]	NiO	5 nm	92 nm
Cho et al. [14]	NiO	10–20 nm	92 nm
Cuevas et al. [31]	Ni(OH)_2_	5 nm	40 ± 1.5 nm
Di Bucchianico et al. [32]	NiO	<50 nm	750 nm
Duan et al. [33]	NiO	<50 nm	306 ± 2 nm
Dumala et al. [34]	NiO	15.6 ± 2.59 nm	169 ± 17.1 nm
Dumala et al. [35]	NiO	13 ± 3.0 nm	111 ± 25.9 nm
Dumala et al. [36]	NiO	12.9 ± 3.4 nm	111 ± 25.9 nm
Dumala et al. [37]	NiO	17.94 ± 3.48 nm	285.9 ± 19.6 nm
Fujita et al. [38]	NiO	10–20 nm	59 nm
Gillespie et al. 2010 [22]	Ni(OH)_2_	5 nm	40 ± 1.5 nm
Gutierrez et al. [17]	NiO	<20 nm	variable from <100 nm to >1 µm
Horie et al. [39]	NiO	15–35 nm	20–100 nm
Horie et al. [40]	NiO	<100 nm	NR
Horie et al. [41]	NiO	100 nm	74–108 nm
Horie et al. [42]	NiO	20 nm	27–39 nm
Horie et al. [43]	NiO	10–20 nm	NR
Horie et al. [19]	NiO (Green) (Black)	Green: 100 nm Black: 20 nm	Green: (NR) Black: (38–180 nm)
Jeong et al. [44]	NiO	5.3 ± 1.9 nm	210 ± 3.7 nm
Kadoya et al. [45]	NiO	26 nm	54 nm
Kang et al. [46]	Ni(OH)_2_	5 nm	40 ± 1.5 nm
Kang et al. [47]	Ni(OH)_2_	38 nm	38 ± 1.4 nm based on SMPS
Katsnelson et al. [48]	NiO	16.7 ± 8.2 nm	NR
Latvala et al. [21]	NiO	<50 nm	0.7–2.2 nm
Lee et al. [49]	NiO	5.3 ± 0.4 nm	224 ± 11 nm
Liberda et al. [50]	Ni(OH)_2_	5 nm	40 nm
Liberda et al. [51]	Ni(OH)_2_	5 nm	40 ± 1.5 nm
Liberda [52]	Ni(OH)_2_	5 nm	40 ± 1.5 nm
Lu et al. [53]	NiO	10–20 nm	NR
Lu et al. [54]	NiO	10–20 nm	NR
Marzban et al. [55]	NiO	28–32 nm	NR
Minigalieva et al. [56]	NiO	16.7 ± 8.2 nm	NR
Minigalieva et al. [57]	NiO	16.7 ± 8.2 nm	NR
Morimoto et al. [58]	NiO	20 nm	139 ± 12 nm
Morimoto et al. [59]	NiO	19 nm	20–100 nm
Morimoto et al. [60]	NiO	8.41 nm	0.48–8.69 µm
Morimoto et al. [61]	NiO	20 nm	26 nm
Morimoto et al. [62]	NiO	8.41 nm	1.34 µm
Nishi et al. [63]	NiO	20 nm	26 nm
Nishi et al. [23]	NiO	10–20 nm	26 nm
Ogami et al. [64]	NiO	20 nm	Instillation: 26 nm Inhalation: 59 ± 3 nm
Ogami et al. [65]	NiO	27 nm	800 nm
Oyabu et al. [66]	NiO	20 nm	139 ± 12 nm
Oyabu et al. [67]	NiO	19 nm	59.7 nm
Pietruska et al. [68]	NiO	<100 nm	>100 nm
Sager et al. [15]	NiO	Not Specified	486 ± 5.8 nm 694 ± 3.7 nm 221 ± 6.6 nm 102 ± 2.9 nm 3060 ± 13.5 nm 1313 ± 8.4 nm 4460 ± 85.4 nm 490 ± 8.9 nm
Saquib et al. [69]	NiO	25.1 ± 2.1 nm	43.3 ± 2.6 and 226 ± 1.5 nm
Senoh et al. [70]	NiO	20 nm	37–68 nm
Shinohara et al. [20]	NiO	Spherical: 20 ± 8 nm Irregular Spherical: 140 ± 67 nm	Spherical: 49 nm Irregular Spherical: 1600 nm
Siddiqui et al. [71]	NiO	22 nm	151 nm
Sutunkova et al. [72]	NiO	23 ± 5 nm	NR
Yu et al. [73]	NiO	20 nm	NR

SD: standard deviation; NR: Not Reported.

## 5. Overview of Toxicological Endpoints and Considerations for Potential Human Health Effects

The literature search found numerous studies that investigate the potential health effects of oxidic nickel nanoparticles including inflammatory effects in the lung, genotoxic endpoints, cardiovascular effects, and other systemic toxicological effects. A more detailed summary of these toxicological endpoints is provided below. However, it is important to note that when characterizing the relevance of nonclinical studies with potential human health effects, several considerations need to be taken into account. First, a substance must be identified as a hazard for a particular disease endpoint or biological pathway. Second, the dose response relationship needs to be characterized including levels where no response is observed. Finally, a detailed exposure assessment must be performed to properly characterize the dose for the exposure scenario of interest. Taking all of these steps into consideration, one can draw conclusions about the potential health risk for a particular disease endpoint. However, with the lack of quality epidemiological data to support any biological responses to oxidic nickel nanoparticles in humans, researchers are left with extrapolating data from various in vitro and in vivo assays. Limitations associated with extrapolation from in vitro to in vivo responses and translation of in vivo rodent assays to human health effects can have significant implications in the application of the risk assessment process.

### 5.1. Estimation of Human Exposures

When examining both in vivo and in vitro toxicology studies, it is helpful to examine how doses in animal or cellular model systems may translate to the human experience. Oftentimes, toxicology studies are aimed to deliver doses that will cause an anticipated toxicological effect without consideration as to how relevant the doses are to human exposures conditions.

Specifically for oxidic nickel nanoparticles, potential human exposure is most likely to occur during occupational handling via inhalation or dermal contact [74,75,76]. However, no studies were identified that evaluated workers exposures to oxidic nickel nanoparticles. It is well understood that welding byproducts are comprised of high levels of ultrafine metals, including Ni nanoparticles [77]. As such, welders give an opportunity to estimate potential nanoparticle exposures to humans. Two studies that attempted to quantify human exposure to Ni nanoparticles in this population were identified in the existing literature. Cena et al. [77,78] aimed to estimate the amount of nanoparticles to which welders are exposed during common welding tasks in two separate studies. In the studies, nanoparticles determined to be smaller than 300 nm, were measured during gas metal arc welding (GMAW) of mild and stainless steel and flux-cored arc welding (FCAW) of mild steel. Researchers collected Ni-containing nanoparticles emitted during these tasks using a nanoparticle respiratory deposition (NRD) sampler, which is a tool that collects nanoparticles that could be deposited within the respiratory tract. The NRD sampler is equipped with a respirable cyclone and an inertial impactor, which remove particles larger than 10 µm and 300 nm, respectively. Ni-containing nanoparticles smaller than 300 nm, and therefore collected within the sampler, were then analyzed and quantified using inductively coupled plasma mass spectrometry (ICP-MS). It was reported that Ni concentrations collected in the NRD samplers ranged from 10 to 51 µg/m^3^ [77,78].

Without exposure monitoring data, it is difficult to predict the expected exposure concentrations to oxidic nickel nanoparticles. However, it was reported that the UK, German, Netherlands and Australia have proposed occupational limits of 2 × 10^10^ particles/m^3^ or a limit of 0.1 times the current worker exposure limit of the coarse material for carcinogenic, mutagenic, asthmagenic, and reproductive toxicants (CMAR) category of nanoparticles [79,80]. This occupational exposure limit is approximately equivalent to 0.56 µg/m^3^ assuming a particle diameter of 20 nm (one 20 nm particle is approximately 2.8 × 10^−8^ ng) for nickel oxide.

#### Dosimetric Adjustment

Inhalation studies investigated the toxicity of Ni nanoparticles in animals exposed to concentrations ranging from 2.8 to 3720 µg/m^3^ of NiO or Ni(OH)_2_ nanoparticles. Specifically, Morimoto et al. [58] and Oyabu et al. [66] exposed rats to 1 × 10^11^ particles/m^3^ for 6 h per day, 5 days per week, for 4 weeks, and observed a peak in inflammation at 4 days post-exposure while 1 month and 3 month time points returned to baseline levels. The daily dose is approximately equivalent to 2.8 µg/m^3^ NiO (assuming a particle diameter of 20 nm) and was the lowest dose tested in all the studies.

In order to relate rodent inhalation data with human exposures an adjustment was performed using data from Morimoto et al. [58] and Oyabu et al. [66]. Exposure concentration was extrapolated to human doses by applying a dosimetric adjustment factor of 1.92 derived using specific study parameters and particle characteristics as described below. This dosimetric adjustment factor was derived based on differences in exposure regime (8 h for human compared to 6 h for rat studies), differences in rat and human ventilation rates (2.1 L/min for 300 g rat; 20 L/min for reference worker), particle size-specific deposition fraction [estimated from Multiple-Path Particle Dosimetry Model (MPPD) for 20 nm size NiO nanoparticle; 0.41 for humans and 0.0397 for rats], and normalizing factor (based on alveolar surface area of the respiratory tract target tissues) (102 and 0.4 m^2^, respectively, in humans and rats) [81,82].

A human equivalent concentration of 5.5 µg/m^3^ was estimated to correspond to the transient lowest observed effect level (LOEL) (e.g., minimum inflammation that returned to baseline) which is approximately 9.8-fold above the proposed occupational exposure limit for CMAR nanoparticles. Other inhalation studies that investigated the toxicity of oxidic nickel nanoparticles exposed animals to concentrations ranging from 100 to 3720 µg/m^3^ of NiO and Ni(OH)_2_ nanoparticles and, thus, are likely to be several magnitudes greater than the European proposed occupational exposure limit for CMAR nanoparticles [22,31,38,39,45,46,47,50,51,52,59,64,67,72]. Therefore, consideration of equivalent exposures needs to be taken when extrapolating the potential toxicity observed with the animal studies to predicting the potential effects in humans.

In addition to inhalation, there is a potential for humans to ingest oxidic nickel nanoparticles. However, no information is available for human exposure to oxidic nickel nanoparticles by ingestion. Dumala et al. [34] reported that they used a dose of 125 mg/kg as “probable human exposure when accidentally exposed to large amounts of NiO nanoparticle” via ingestion; however, it was unclear how and from what source this anticipated human dose was derived. The Agency for Toxicological Substances and Disease Registry reported that the average daily dietary intake of total nickel (not likely to be nickel nanoparticles) in food ranges between 69 and 162 µg/day or 0.98 and 2.3 µg/kg/day for a 70 kg person [5].

Overall, there is a critical research gap that needs to be addressed to understand the relevancy of the exposure levels used in these studies to expected human exposure. It seems likely that the majority of the studies exposed animals/cells to oxidic nickel nanoparticle concentrations several orders of magnitude greater than what is expected to be physiologically relevant for human exposure scenarios based on limited data.

### 5.2. Considerations for In Vitro and In Vivo Studies When Evaluating Human Health Effects

In vitro studies have some advantages over in vivo animal studies in that these types of studies are generally rapid to perform and may offer insights into the molecular pathways that potentially occur in vivo. However, mechanisms such as changes in uptake processes, metabolic handling, and efficiency of cellular repair mechanisms can all be affected by dose and dose rate when evaluating in vitro and in vivo data for relevancy towards in vivo mechanisms [83]. For example, high concentrations of particles may initiate cellular necrosis due to an increase in reactive oxygen species (ROS) in vitro but that mechanism may have limited importance in vivo where relevant exposure levels do not overwhelm the antioxidant system and initiate such biological responses. Therefore, careful considerations must be made when extrapolating high dose in vitro and in vivo data. Furthermore, in vitro assays are unable to replicate the dynamic conditions associated with tissue-specific clearance mechanisms and heterogeneous cell populations involved with different biological responses. For example, numerous in vitro studies utilized various cancer cell lines (e.g., A549, HeLa, HepG2, etc.) that have genetic mutations to allow for better survival under in vitro culture conditions, but have limitations when studying molecular pathways in an attempt to characterize potential biological responses in vivo. In addition, many in vitro studies utilize monoculture systems which lack cell–cell interactions that may be important for the development or mitigation of toxicity. As such, in vitro conditions are also unable to replicate chronic exposure scenarios that are more relevant to some types of human exposures.

Dose metrics for in vitro assays do not typically take into account the relevance to in vivo exposure scenarios. Nanoparticles’ physical and chemical properties can affect the transport of particles to cells in vitro [16,84,85]. Properties such as size, shape, and density of nanoparticles along with media density and viscosity can affect settling rates, diffusion, agglomeration, and ultimately the delivered dose of nanoparticles to each cell. Ahmad Khanbeigi et al. [84] defined administered dose as the particle number, mass, or surface area per volume of suspension media; delivered dose as the particle number, mass, or surface area to reach the cell monolayer; and cellular dose as the particle number, mass, or surface area to be internalized (or firmly attached) into the cell monolayer. Their study found that the use of the In vitro Sedimentation and Diffusion and Dosimetry Model (ISDD) provided an accurate interpretation of acute in vitro results when the data was normalized to delivered dose. Methods such as fluorescent or radioactive labeling have been used to measure delivered dose for other nanomaterials [16]. Additionally, numerous analytical methods such as mass spectrometry, inductively coupled plasma mass spectrometry, and liquid chromatography mass spectrometry can be used to measure delivered dose. While Cohen et al. [85] did not specifically study oxidic nickel nanoparticles, the authors examined the importance of particle kinetics in various physiological media for several other metal oxide nanoparticles and found that particle kinetics and property transformations have significant effects on in vitro dosimetry and need to be considered when interpreting cellular toxicological information. Teeguarden et al. [16] showed that while typically equal mass concentrations imply equal doses, in fact, for nanomaterials, the delivered dose to the cells differed by orders of magnitude depending on the nanomaterials composition and size. Further, agglomeration of nanoparticles has been shown to affect sedimentation rates and ultimately the actual dose each cell encountered [16], but the majority of in vitro studies did not characterize sedimentation rate under various biological conditions. Some studies have attempted to avoid agglomeration by dispersion. However, Gutierrez et al. [17] reported that the dispersion media differentially affected nanoparticle physicochemical properties and toxicity. Further, the authors concluded that studies using re-suspension methods of exposure of nanoparticles may cause variation of particle characteristics and transport processes which can lead to inconsistent, and even misleading, biological results that are not representative of in vivo conditions.

In vitro studies have not related cellular and in vivo conditions, such as dose per surface area of cells compared with dose per surface area in a human lung. Although limitations still exist with extrapolation, this type of analysis could provide a general benchmark for which in vitro doses may be somehow correlated in vivo.

Taken together, incorporation of particokinetics and principles of dosimetry are needed when evaluating any in vitro assay for comparisons across other particle types, other in vitro assays, or when extrapolating to in vivo responses. Despite the evidence suggesting that it is necessary to study the dosimetry associated with the nanoparticle exposure, the majority of current literature regarding oxidic nickel nanoparticles did not take in vitro dosimetry into consideration and may explain some of the differences in biological responses. Further considerations when analyzing in vitro data include: (1) sedimentation and actual dosing to cells as described above [16,84,85], (2) extrapolation of surface area of cells in a cultured dish to the lung, (3) diffusion, and (4) different dosing due to varying solubility and dose metrics, particularly when comparing nano- vs. micron-sized particles. Nonetheless, these in vitro systems have been utilized for decades to examine toxicity endpoints such as cytotoxicity, apoptosis, ROS generation, and inflammatory pathways. While data from appropriately designed in vitro assays can be potentially useful for hazard identification and studying aspects of certain molecular pathways, this kind of data remains limited for human health risk assessment. Therefore, this evaluation primarily focuses on the in vivo studies (in vitro studies are outlined in Supplemental Table S2), although some in vitro studies are discussed for certain endpoints, such as genotoxicity.

In vivo rodent assays hold many advantages over in vitro assays and are more relevant to potential human responses. Dynamic processes such as tissue deposition and clearance in vivo can provide more relevant exposure scenarios in contrast to in vitro systems. However, many limitations still exist. Unphysiological bolus dosing such as intratracheal instillation, pharyngeal aspiration, and intramuscular injection are not relevant to potential human exposure scenarios and limit the interpretations of these results. In contrast, physiological inhalation exposure is relevant to human experiences and provides data with fewer limitations. Studies comparing the pulmonary effects of inhaled versus instilled particles have shown that at the same retained dose the deposition and retention pattern, as well as the subsequent cellular responses and pathology can be very different between the two delivery methods. In fact, often times pathological responses observed with bolus dosing by intratracheal instillation are not replicated in inhalation studies with similar target dosing amounts [86,87,88,89]. Furthermore, various species-specific factors such as deposition rate, clearance, and tissue reactions may limit aspects of in vivo studies when extrapolating from animal data to potential human health effects. Nonetheless, in vivo data can aid in the identification of potential pathways that may play a role in biological responses in humans.

### 5.3. Dose, Deposition, and Clearance

Risk characterization involves (1) identification of the substance as a hazard, (2) characterization of dose response relationship, (3) assessment of exposure to the substance and (4) risk characterization. After taking these into consideration, one can characterize the potential health risk associated with exposure to a particular substance [90]. Thus, to understand the risk, it is necessary to consider dosimetric extrapolation and dose-metrics when deriving a human equivalent concentration, and consider occupational or environmental exposure limits when performing human risk extrapolation modeling based on results of rodent inhalation studies. Limitations associated with extrapolation and translation of in vivo rodent assays to human health effects can have significant implications in the application of the risk assessment process. With proper experimental design, an analysis can be performed to determine the dose associated with a no-observed-adverse-effect level (NOAEL) and lowest-observed-adverse-effect level or concentration (LOAEL or LOAEC) and then apply uncertainty factors for animal to human extrapolation. Other methods such as the benchmark dose (BMD) approach can also be utilized to further understand threshold levels below which, no response is expected to occur. However, most oxidic nickel nanomaterial studies tested a limited number of doses and few studies identified NOAELS and LOAELs. Of the 17 studies that evaluated the toxicity associated with NiO nanoparticles via the inhalation route of exposure, only seven studied multiple concentrations [22,31,39,47,51,59,67]. Furthermore, only three studies tested three concentrations to allow for an analysis of the dose response curve [22,31,51]. Out of the publications that reported testing multiple concentrations, only two studies determined NOAELs [22,59] and the remaining reported LOAELs.

Although inhalation is relevant to human exposure, several considerations still need to be made. First, the exposure of each rodent in vivo study will need to be related to potential human exposure levels. Most inhalation studies used various occupational exposure limits as a benchmark for their dose metrics. The Permissible Exposure Limit (PEL) of 1000 µg/m^3^ as set by the Occupational Safety and Health Administration (OSHA) for micron-size nickel exposures is typically referenced. The American Conference of Governmental and Industrial Hygienists’ Threshold Limit Value (TLV) of 200 µg/m^3^ (as inhalable aerosol fraction) for insoluble nickel compounds such as NiO and 1500 µg/m^3^ for metallic nickel are also referenced. The concentrations of NiO nanoparticles in the experimental rodent studies ranged from 100 to 3720 µg/m^3^ for inhalation studies. However, relating the exposure dose and duration of exposure in rodent assays to the human experience is more complicated than a benchmark to occupational exposure limits. Species-specific factors that determine deposition factors and clearance rates will have an impact on biological responses. Future studies are needed to better model the deposition potential of NiO nanoparticles in humans compared with various rodent assays. For instance, the MPPD can be used to extrapolate between animals and humans to better understand relevancy of exposure metrics in each animal assay [91].

Respiratory tract deposition and clearance were described in several rodent experiments. Oyabu et al. [66] found that 29 µg of nickel was retained in the lungs of rats 4 days after inhalation to NiO nanoparticles for 4 weeks at a concentration of 1.0 × 10^5^ particles/cm^3^. A biological half-time of 62 days (approximately 2.1 months) was calculated. In a later study, Oyabu et al. [67] reported that 40.0, 24.6, and 19.0 µg of NiO was retained in the lungs after 3 days, 1 month, and 3 months, respectively, post inhalation of 0.32 mg/m^3^ NiO nanoparticles for 4 weeks; a biological half-time of 2.9 months was estimated. Additionally, the authors reported that rats exposed to a higher concentration of 1.65 mg/m^3^ of NiO nanoparticles via inhalation retained NiO nanoparticles in the lung at a concentration of 132.5, 130.0, 92.4 µg at 3 days, 1 month, and 3 months after exposure; a biological half-time of 5.2 months was calculated. Likewise, intratracheal instillation of 0.2 mg NiO nanoparticles led to a retention of 136.4 to 59.4 µg NiO nanoparticles 3 days to 6 months after instillation; a biological half-time of 4.9 months was calculated. The authors reported that intratracheal instillation of 1 mg NiO nanoparticles led to a retention of 738.1 to 465.5 µg NiO nanoparticles on day 3 to month 6 after exposure; a biological half-time of 9.5 months was estimated [67]. Further, Shinohara et al. [20] estimated the pulmonary clearance rate for several NiO nanoparticles using a one-compartment model. The estimated half-time for spherical NiO nanoparticles were 310, >690, and 410 days for exposure to a one-time concentration of 0.57, 1.9, and 5.8 mg/kg administered via intratracheal instillation, respectively. Irregular shaped nanoparticles had an estimated half time of 59, 170, >690 days for doses of 0.47, 2.0, 5.8 mg/kg. It should be noted that the concentrations used in the rodent experiments are not representative of expected human exposure.

In contrast to the NiO nanoparticles, a reported deposition rate of 17 to 24% was determined and a half-time of approximately one day was calculated by Gillespie et al. [22] for Ni(OH)_2_ nanoparticles. Briefly, mice were exposed for 4 h to nominal concentrations of 100, 500, and 1000 mg/m^3^ of Ni(OH)_2_ nanoparticles via whole body inhalation. Potential reasons for differences in the half-time between the NiO and Ni(OH)_2_ nanoparticles include animal species variances, particle chemical form, particle size, solubility, and exposure duration. The Ni(OH)_2_ nanoparticles used in Gillespie et al. [22] were almost all dissolved (discussed further in Section 4) and it is likely the high solubility of Ni(OH)_2_ nanoparticles drove the clearance, compared to the NiO nanoparticles. Indeed, the values by Oyabu et al. [66] were more similar to the half-time calculated for micron-sized NiO particles (approximately 3 months) [22,66].

## 6. Overview of Lung Inflammation

Inhalation is the primary route of potential exposure to oxidic nickel nanoparticles in occupational or industrial settings. Thus, there is an interest in understanding the potential risk of pulmonary inflammation and toxicity. Inflammation encompasses a complex, organized assembly of responses to toxicants, pathogens, cell damage, or irritants. Although acute inflammation is beneficial and necessary for the body’s natural defense against foreign material such as pathogens, chronic activation of the inflammatory pathway in the lungs can lead to more serious health effects including fibrosis. However, several factors can determine the chronic nature of the inflammatory response including the stimulant and exposure metrics along with tissue-specific responses that dictate severity and potential to resolve the inflammation. Many studies investigated the effect of NiO nanoparticles via intratracheal instillation and whole body inhalation in in vivo studies (Table 2) and in vitro studies utilizing lung cell lines (Supplemental Table S2). The strengths and weaknesses of in vitro and in vivo studies were considered (discussed in Section 5.2) when selecting and detailing the key studies.

The acute versus chronic nature of the inflammatory response can be observed in a series of studies that instilled NiO nanoparticles into rats at doses ranging from 100 to 2000 µg/rat with a follow-up time ranging from 3 days to 12 months [23,39,59,60,61,62,63,67]. Doses of 100, 200, and 1000 µg/rat caused an increase in inflammation as characterized by BALF cell counts, lung weight, BALF protein and phospholipid concentration that increased from 3 days to 3 months post-instillation but returned to baseline levels after 6 months [23,61,63,67]. Cytokine levels were also assessed and displayed transient effects at various doses displaying the acute nature of the inflammatory response. An additional study by Ogami et al. [64] found that the inflammatory response peaked at 6 months and subsided at 12 months post-instillation of NiO nanoparticles at a dose of 200 µg/rat. This data suggests that the inflammatory response induced by NiO nanoparticles was acute and transient in nature. In later studies by the same authors, a dose of 1000 µg/rat was used to observe a chronic response characterized by significantly increased cell count and cytokine levels up to 6 months post-instillation [40,59,60,62]. However, responses longer than 6 months were not characterized and therefore, the extent of this chronic response remains in question. If assayed for a longer period of time, it is possible that the inflammatory response may recover similar to previous experiments.

In an even higher dose, Shinohara et al. [20] reported that intratracheal instillation of a one-time dose of 6000 µg/kg of NiO nanoparticles (20 nm) to rats led to an increase in pulmonary inflammation at 13 weeks post-administration; however, extended recovery time was not assayed. Ogami et al. [65] instilled 2000 µg of NiO nanoparticles per rat and observed an increase in BALF cell counts up to six months post-instillation; however, once again, longer recovery time was not assayed. Interestingly, there were data to suggest that the inflammatory response to NiO nanoparticles was recovering at the 6 month follow-up time point as PMN infiltration decreased over time. In comparison, the inflammatory response to crystalline silica increased over time. These data suggest that even with a dose as high as 2000 µg of NiO nanoparticles, the inflammatory response may be acute in nature with the potential to resolve over longer periods of time.

Cho et al. [13] also observed dose-dependent, transient acute inflammation after instillation of a one-time dose of NiO nanoparticles starting at 50 cm^2^/rat (54.5 µg/rat). Inflammatory responses increased 24 h post-exposure but diminished at 4 weeks post-exposure. When NiO nanoparticles were instilled into rats, the lymphocyte response was diminished but still significant compared with vehicle control. Furthermore, fibrosis was not observed at 4 weeks in the NiO nanoparticle-exposed rats while zinc oxide and copper oxide nanoparticles were capable of inducing a fibrotic response. However, other data by Cho et al. [12] displayed a delayed type-hypersensitivity (DTH)-like mononuclear inflammation, characterized by pulmonary alveolar proteinosis (PAP), epithelial proliferation, lymphocytic foci, and granulocytic infiltration in the airspace 4 weeks post-exposure under a similar dosing protocol to 150 cm^2^/rat (163.5 µg/rat). These data demonstrate that it is possible for NiO nanoparticles to induce lymphocytic infiltration through TH1-related cytokine expression including IFN-γ when instilled into rats at a dose of 163.5 µg/rat. However, it should be noted that both studies used NiO nanoparticles that were dispersed in rat serum, as compared to water or PBS in other studies, to prevent large agglomeration of particles [12,13]. Thus, it is possible that the solubility of nickel from NiO nanoparticles bound to the serum proteins and can cause haptenic responses that lead to DTH-like pathology. Future studies may attempt to characterize the role of solubility in inducing DTH and other inflammatory pathways in animal studies; however, the current data set does not provide insight into the parameters that control this biological outcome.

Bai et al. [28] studied the effect NiO nanoparticles (20 nm) had on pulmonary inflammopathology 1 to 28 days after intratracheal instillation of 10 to 100 µg NiO nanoparticles to mice. The authors reported that exposure to 20 µg NiO nanoparticles led to increased LDH and 8-OHdG levels at 24-h follow-up time and exposure to 10 and 20 µg NiO nanoparticles led to increased total pulmonary protein levels and IL-6 levels. However, there were no significant differences in these levels, except for 8-OhdG which was still increased, at 28-days post-exposure. The authors reported that inflammation scores in lung, as determined by single-photon emission CT analysis, were dose-dependent and intratracheal instillation of 100 µg NiO nanoparticles induced a response that was scored as severe. Additionally, lung parenchyma inflammation and small airway inflammation was observed only in the left and right subsegments of the secondary bronchial bifurcation and the end of the secondary bronchial bifurcation, as observed via Chest CT, 1 day after exposure while lung parenchyma inflammation and small airway inflammation was observed in the whole lung 7 days after exposure.

Lu et al. [53] studied the effect NiO (10–20 nm) nanoparticles had on ROS potential in both in vivo and in vitro systems and, thus, the correlation of in vitro results to in vivo pro-inflammatory activity. NiO nanoparticles were able to cause in vivo inflammation characterized by a significant increase in total polymorphonuclear leukocytes (PMNs); however, ROS generation in vitro did not correlate with inflammation activity in vivo.

Taken together, the intratracheal instillation studies demonstrate an ability of NiO nanoparticles to induce acute lung inflammation under certain exposure conditions in rats and mice. However, exposure to large doses of NiO nanoparticles that are not relevant to human exposures was necessary to induce responses in rats. The studies did not investigate the effect of an extended follow-up time that may allow a complete recovery from the exposure. Further, these responses may not necessarily be specific to NiO nanoparticles as any particle type may elicit non-specific responses at high concentrations. Intratracheal instillation ensures that the majority of administered particles reaches the lung while inhalation requires lung burden analysis to understand the administered dose. However, instillation requires extremely low volumes with high concentrations of particles that are administered in a fraction of a second in bolus form which is not physiologically representative of the low-dose rate that occurs by inhalation. As such, a bolus injection, such as instillation, delivers high doses of NiO nanoparticles that can cause non-specific responses such as lung inflammation and limits the interpretation of these data when extrapolating to potential human health effects.

Other evidence for the acute nature of the inflammatory response was observed when rats were exposed to NiO nanoparticles via whole body inhalation [39,45,58,59,64,66,67]. The studies described an acute inflammatory response followed by recovery to baseline levels. Ogami et al. [64] dosed rats with 200 µg/m^3^ (9.2 × 10^4^ particles/cm^3^) for 4 weeks and found that the inflammatory response peaked at 1 month-post exposure and decreased to baseline levels at 3 months. Morimoto et al. [58] and Oyabu et al. [66], exposed rats to NiO nanoparticles at a concentration of 1.0 × 10^5^ particles/cm^3^ (approximately 2.8 µg/m^3^) for 4 weeks and observed a peak in inflammation at 4 days post-exposure while 1 month and 3 month time points returned to baseline levels. Morimoto et al. [59] and Horie et al. [39] dosed rats with NiO nanoparticles at 1650 µg/m^3^ for 4 weeks and noted a significant elevation in total cell count, and CINC-1 on day 3, and an increase in HO-1 concentration over the first month; all of the inflammatory responses returned to baseline by six months. Kadoya et al. [45] reported that rats exposed to 0.2 mg/m^3^ NiO nanoparticles (26 nm) via whole-body inhalation for four weeks had elevated concentrations of phospholipids, total protein, and surfactant-specific protein in the BALF, an increased PMN count, and infiltration of macrophages into the lung alveolar space and interstitium following 3 and 30 days. The levels declined from 3 days to 1 month post-exposure; further, the levels were not elevated at 3 months post-exposure. These data demonstrate that the inflammatory response to NiO nanoparticles in rats can be characterized as acute in nature and suggests that ample clearance and recovery is occurring in the lung at these particle concentrations.

Sutunkova et al. [72] examined the effects of 1.0 mg/m^3^ NiO nanoparticles on rats after being inhaled for 4 h per day for 5 days via nose-only inhalation. The authors reported that there was an increase in the BALF total cell counts and number of alveolar macrophages and neutrophil leukocytes compared to the control animals at 24 h after the last exposure. At 3 weeks after the last exposure, the BALF total cell count and number of alveolar macrophages was still statistically significantly increased, but was decreased compared to the levels at 24 h; the initial neutrophilic response was no longer present. In a follow-up chronic study, Sutunkova et al. [72] exposed rats to 0.23 mg/m^3^ NiO nanoparticles for 4 h per day, 5 times per week, for 3, 6, or 10 months via nose-only inhalation. The authors reported that the rats exhibited a statistically significant increase in BALF total cell counts due to the recruitment of alveolar macrophages and neutrophil leukocytes after all exposure lengths; the response peaked at 3 months and decreased for the 6 and 10 month exposure groups. In addition, the authors reported that there was increased activity of the BALF supernatant’s enzymes including increased γ-gutamyl transferase, amylase, lactate dehydrogenase, alkaline phosphate, and aspartate aminotransferase levels. Histological evidence did not show any cellular fibrotic nodules or thickening of interalveolar septae; however, there was evidence of the septae becoming thinner or even destroyed while the reticulin framework was not coarser than the control rats.

Kang et al. [46] studied the effects on lung inflammation after inhalation by ApoE^−/−^ mice of Ni(OH)_2_ nanoparticles (5 nm) at a concentration of 100 µg/m^3^ for 1 week and 5 months. Cell counts, neutrophil influx, and protein levels increased significantly for both time points in BALF. Cytokine and chemokine profiles displayed mixed results depending on time point and marker. Histopathology displayed mild to moderate inflammation. Levels of HO-1, but not other antioxidant enzymes, were upregulated in lung for both time points. Gillespie et al. [22] utilized short (103.2, 565.0, and 1204.0 μg/m^3^ for 4 h) and long (124.0, 129.3, and 124.5 μg/m^3^ for 5 h/day, 5 days/week, for 1 week, 3 months, or 5 months) durations of exposure to characterize the acute versus chronic effects of Ni(OH)_2_ nanoparticles in mice that were exposed via whole body inhalation. Low exposures (103.2 µg/m^3^) had no effect on inflammatory responses after only a short term exposure duration (4 h). However, long term exposure to low levels (124–129.3 µg/m^3^) for five months presented mild inflammatory infiltrates in the pulmonary parenchyma mostly comprised of lymphocytes along with significant increases in various chemokine levels. These data indicate that long term exposure may initiate inflammation in mice; however, there was no evidence of fibrosis and the inflammatory response was characterized as mild even after 5 months of exposure. Furthermore, recovery after a longer follow-up beyond 24 h was not assessed. It is possible that these effects would return to baseline levels if assayed at longer follow-up times, or possibly continue with ongoing exposure.

Taken together, the inhalation studies demonstrate that oxidic nickel nanoparticles cause an acute inflammatory response to rats and mice at relatively large doses. However, there is a lack of evidence suggesting that oxidic nickel nanoparticles caused chronic damage. In fact, it was reported that rats were able to clear the nanoparticles and undergo recovery in the lung at particle concentrations up to 1650 µg/m^3^. As with the instillation studies, careful considerations need to be taken into account when interpreting relevance of animal doses, and particle characteristics in comparison to potential human exposure scenarios.

Horie et al. [39] and Morimoto et al. [59] compared lung inflammation after intratracheal instillation or whole body inhalation of NiO nanoparticles. They noted that the pulmonary inflammation observed in the intratracheal instillation studies was not observed in the inhalation studies. Horie [39] and Morimoto et al. [59] estimated that the NiO lung burden was similar in rats dosed with NiO nanoparticles after intratracheal instillation and inhalation. They reported that the observed “general” pulmonary oxidative stress response was similar between the inhalation and instillation studies though there were differences in the early phases of the oxidative stress and specifically inhalation caused milder oxidative stress. Few other data were comparable for the relationship between instilled versus inhaled NiO nanoparticles. While Ogami et al. [64] compared the lung inflammation after both instillation and inhalation exposure to nanoparticles, NiO nanoparticles were used as a positive control for studying the effects of fullerene particles and there were only histopathology results available. The authors reported that the NiO nanoparticles caused inflammatory changes via both inhalation and instillation route of exposure; however, the inflammatory response had decreased by 3 months for rats exposed to NiO nanoparticles via inhalation, while the inflammatory response observed after exposure via instillation persisted until 12 months. By contrast, Mizuguchi et al. [90] compared instillation and inhalation of NiO nanoparticles and reported that a comparable inflammatory response via inhalation was observed at pulmonary deposition amount equivalent to one tenth of the intratracheal instillation dose. Taken together, the relevance of performing instillation or pharyngeal aspiration to mimic potential lung responses after inhalation of NiO nanoparticles remains limited.

## 7. Overview of Systemic Toxicological Endpoints

Numerous rodent studies investigated the effect of NiO nanoparticles on systemic endpoints, such as mortality, the cardiovascular system, and other organs (Table 3). Additionally, in vitro studies investigated the effect of NiO nanoparticles on cells associated with various organs and systems such as the skin, breast, hematopoietic system, and others (Supplemental Table S2).

Shinohara et al. [20] reported that rats dosed with 0.67–6.0 mg/kg NiO nanoparticles via intratracheal instillation had statistically significant higher NiO burdens in most of the thoracic lymph nodes compared to the control animals; the authors reported that the response was dose- and time-dependent for rats exposed to spherical NiO nanoparticles and irregular shaped NiO nanoparticles. Further, the author reported there was an increased NiO burden in the liver of rats dosed with NiO nanoparticles compared to control animals; however, there was no clear dose- or time-dependency. Dumala et al. [35] studied the total Ni content in various organs after oral gavage of 125–500 mg/kg NiO nanoparticles to rats. They showed that the liver accumulated more nickel, followed by the brain, kidney, and finally the spleen. Taken together, deposition and clearance are potentially affected by assay variables such as methodology, species, dose, and nanoparticle chemical form and size. Future studies may better describe how deposition and clearance are affected by each of these variables.

### 7.1. Mortality

Mortality was included in only one study. Dumala et al. [36] reported no mortality to rats exposed to 200 mg/kg/day NiO nanoparticles daily via oral gavage for 28 days but did show symptoms such as dullness, irritation, and distress. Since none of the other studies reported mortality, it can be assumed that there was not an increased incidence of mortality after exposure to oxidic nickel nanoparticles. Further, the evidence suggests that, at the doses tested in animals, there is limited evidence that oxidic nickel nanoparticle exposure can induce severe health effects, such as death, via any route of exposures.

### 7.2. Cardiovascular

There is limited evidence of oxidic nickel inducing cardiovascular effects [5]. However, it has been reported that high concentrations of nickel in air pollution (PM2.5, comprised of complex nickel oxides and sulfate) may contribute to cardiovascular effects [92]. As such, the effects Ni(OH)_2_ nanoparticles on the cardiovascular system has been studied in several publications.

Cuevas et al. [31] studied the vascular effects in mice exposed to 100, 150, and 900 µg/m^3^ Ni(OH)_2_ nanoparticles (5 nm) for 5 h/day for 1, 3, and 5 days via whole body inhalation. At 24-h post exposure, carotid arteries from mice exposed to all concentrations of Ni(OH)_2_ nanoparticles displayed differences in graded doses of phenylephrine-induced contractile responses and acetylcholine-induced vasorelaxation responses compared with mice exposed to filtered air. Some responses increased with dose but the 5-day low dose did not differ significantly from 3-day low dose for vasocontraction. It is important to note that no other particles were tested for comparison of responses including nonspecific particle effects. Other cardiovascular effects were studied via whole body inhalation of mice to Ni(OH)_2_ nanoparticles (40 nm) [31,50,51,52]. In the study by Liberda et al. [51], mice were exposed to low (100 µg/m^3^ for 5 h/day for 5 days), moderate (700 µg/m^3^ for 5 h/day for 3 days), and high (1200 µg/m^3^ for 5 h/day for 2 days) concentrations and endothelial cell effects were examined at 1 day post-exposure. Significantly lower % of bone marrow endothelial progenitor cells were observed for moderate and low exposures (high dose was not tested). Cell function tests displayed impaired endothelial progenitor cell chemotaxis and tubule formation after high levels of exposure (lower doses not tested). No significant difference was observed in cellular signaling pathways involved in endothelial cell mobilization, homing, and differentiation. In similar experiments, Liberda [52] and Liberda et al. [50] exposed mice to 500 µg/m^3^ of Ni(OH)_2_ nanoparticles for 5 h and examined cardiovascular responses including endothelial cell effects at 30 min and 12 h post-exposure. The population of bone marrow and circulating progenitor/endothelial cells increased after Ni(OH)_2_ exposure. Differences in cellular signaling pathways were reported to relate to a decrease in cellular homing and an increase in “stickiness” after Ni(OH)_2_ exposure. MCP-1 levels in the aorta were downregulated after Ni(OH)_2_ treatment, suggesting a decrease in chemotaxis signaling. Endothelial progenitor cells from Ni(OH)_2_ exposed mice displayed significantly reduced cellular function for chemotaxis and tube formation. Proteomic analysis of plasma found that transferrin was downregulated and several antioxidants were upregulated. Additionally, Kang et al. [46] studied the effects on cardiovascular inflammation after inhalation by ApoE^-/-^ mice of Ni(OH)_2_ nanoparticles (5 nm) at a concentration of 100 µg/m^3^ for 1 week and 5 months. Levels of HO-1 but not other antioxidant enzymes were upregulated in the heart and aorta tissue for both time points. Cytokine and chemokine levels did not increase in systemic organs after 1 week; however, Ccl-2 and IL-6 were significantly upregulated in the heart after 5 months. Furthermore, mitochondrial DNA damage was observed in the aorta while areas of plaque lesions, Ccl-2, Vcam-1, and Cd68 levels in the aorta also increased after 5 months of exposure.

Overall, the chronic nature of these cardiovascular responses were not well characterized and need to be further studied. While there was evidence of cardiovascular inflammation, contractile and vasorelaxation responses, and effects on endothelial progenitor cells after inhalation of Ni(OH)_2_ nanoparticles, the response were not assessed past the 24 h follow-up time point. Furthermore, the dose response was not well characterized. Further testing of oxidic nickel nanoparticles, including with NiO nanoparticles, long-term follow-up, and testing with longer exposure durations, is needed to better understand any cardiovascular effects.

### 7.3. Other Systemic Effects

The effects of NiO and Ni(OH)_2_ nanoparticles on organ damage was evaluated based on changes to biochemical and functional indices, along with changes in histopathology, in rats administered nanoparticles via inhalation, intratracheal instillation, oral gavage, and intraperitoneal injection. Most studies evaluated the acute toxicity of these nanoparticles while only one study evaluated the response from a chronic exposure.

In an initial experiment, Sutunkova et al. [72] exposed rats to 1.0 mg/m^3^ NiO nanoparticles for 4 h per day for 5 days via nose-only inhalation. The authors reported that there was a significant increase in liver weight, elevated levels of lactate dehydrogenase, evidence of leukocytosis, systemic inhibition of the oxidation-reduction energy metabolism, enhanced lipid peroxidation as measured by increased concentrations of malodialdehyde, and stimulation of erythropoiesis at 24 h post-exposure. In a follow-up chronic study, Sutunkova et al. [72] exposed rats to 0.23 mg/m^3^ NiO nanoparticles for 4 h per day, 5 times per week, for 3, 6 or 10 months via nose-only inhalation and reported the observed effects at 24 h post-exposure. The authors reported that NiO nanoparticle exposure caused erythropoiesis stimulation, an elevated hemoglobin content, an increased erythrocyte count with elevated proportions of reticulocytes, and an elevated hematocrit after 3 months. However, except for an elevated proportion of reticulocytes, these levels had returned to baseline for rats exposed to NiO nanoparticles for 6 and 10 months. Further, there was no noted evidence of increased inhibition processes in the CNS. While the authors reported statistically significant changes in lung, kidney, and liver weights following exposure to NiO, they noted that there were no noticeable changes in the weight of the liver, spleen, and kidneys (per 100 g body mass), or in the indices of liver and kidney functioning. Specifically, the authors reported that there was a statistically significant increase in lung and kidney mass after 10 months of exposure, and a statistically significant decrease in liver mass after 6 months of exposure. However, there were appreciable pathological changes in the histological structure of the liver, as seen by increased number of akaryotic hepatocytes, binucleated hepatocytes, and Kupffer cells; spleen, as seen by increased diameter of the follicle; and kidney, as seen by brush border loss in proximal convoluted tubules at 3 months only and full epithelial desquamation at 10 months.

Yu et al. [73] exposed rats to 0.015, 0.06, or 0.24 mg/kg NiO nanoparticles via intratracheal instillation twice a week for six weeks. The authors reported that rats exposed to 0.24 mg/kg NiO nanoparticles had significantly increased liver wet weight and coefficient to body weight, and had liver pathological changes, such as cellular edema, hepatic sinus disappearance, and binucleated hepatocytes. The authors suggest that the liver toxicity induced by NiO nanoparticles may be associated with nitrative and oxidative stress.

Kang et al. [46] reported that HO-1 mRNA levels were upregulated in the lung, spleen, heart and aorta tissues and liver SAP mRNA levels were increased in ApoE^-/-^ mice that inhaled Ni(OH)_2_ at a concentration of 100 µg/m^3^ for 1 week and 5 months. Further, cytokine and chemokine mRNA levels did not increase in systemic organs after 1 week; however, Ccl-2 and IL-6 mRNA levels were significantly upregulated in the heart and spleen after 5 months. TNF-α was also significantly upregulated in the spleen after 5 months of exposure. Senoh et al. [70] reported no damage to the liver, kidney, spleen, brain, based on organ weight and histopathology, and noted no significant changes in the hematology and blood biochemistry for rats that were intratracheally instilled with 2 mg/kg NiO nanoparticles as either a one-time dose or a divided dose.

Dumala et al. [34] and Dumala et al. [35] reported organ damage, based on dose-dependent changes to biochemical indices, to the brain, liver, and kidney after oral gavage of a one-time dose of 5–2000 mg/kg of NiO nanoparticles to rats. Additionally, 2000 mg/kg NiO nanoparticles caused liver tissue damage and focal areas of necrosis in the liver; however, there was limited to no significant changes in spleen, heart, brain and kidneys. In a follow-up study, Dumala et al. [36] reported organ damage to liver, kidney, spleen, brain, heart, and stomach, as evidenced by significant alterations to biochemical indices and histopathology, along with alterations to hematological parameters after daily exposure to 200 mg/kg NiO nanoparticles via oral gavage for 28 days. Ali et al. [27] reported dose-dependent organ damage to the liver and kidney, based on significant changes to biochemical parameters, along with alterations to hematological parameters, after exposure to 500 or 1000 mg/kg NiO nanoparticles via oral gavage for 14 days.

Katsnelson et al. [48] reported that intraperitoneal injection of 0.25–0.5 mg NiO nanoparticles to rats caused significant changes to biochemical and functional indices associated with the kidney, spleen, brain and liver. The authors reported that seven of these indices were dose-dependent. Additionally, Minigalieva et al. [56] reported that 500 µg NiO nanoparticles administered via intraperitoneal injection three days per week for six weeks caused a statistically significant adverse deviation in functional and biochemical indices associated with the kidney, liver, spleen in rats.

Marzban et al. [55] administered intraperitoneal injections of 10 to 50 mg/kg NiO nanoparticles to rats for 7 days. Rats exposed to all doses of NiO nanoparticles had a significant decrease in GSH levels. The authors reported that rats exposed to 25 and 50 mg/kg NiO nanoparticles showed a dose-dependent significant increase in Glutathione-S-Transferase and catalase activity. In addition, rats exposed to 50 mg/kg NiO nanoparticles showed a significant decrease in total antioxidant capacity and a significant increase in MDA levels. Further, histopathological changes, including necrosis, hyperemia, gliosis, and spongy changes, in the brain were observed for rats dosed with NiO nanoparticles in a dose-dependent manner.

In general, there were conflicting results regarding whether oxidic nickel nanoparticles caused damage to the liver, kidney, and brain based on changes to biochemical indices. Further, the biological significance of the changes to the various biochemical parameters is not fully understood and needs to be further investigated. In general, there was limited histopathological evidence showing organ damage after acute exposures to NiO and Ni(OH)_2_ nanoparticles. One study observed histopathological changes to the brain after intraperitoneal injection of NiO nanoparticles [55]; however, other studies that administered NiO nanoparticles via oral gavage did not report any histopathological changes in the brain [34,35]. Further research needs to be done to understand whether this effect is observed after inhalation route of exposure. Additionally, while there was evidence of mild to severe damage to organs after daily oral gavage of NiO nanoparticles for 28 days, this was only seen at significantly high doses (200 mg/kg/day). As such, most of the studies investigated only the acute nature of these various organ toxicities and did not investigate the potential chronic effects or recovery. Only one study investigated the chronic nature of the effects that NiO nanoparticles had on systemic toxicity when exposed via inhalation (0.23 mg/m^3^ NiO nanoparticles for 4 h per day, 5 days per week for 3, 6, and 10 months) [72]. The authors reported toxicity to the liver and kidneys, as indicated by changes to histopathological indices; however, it is unclear the effect NiO nanoparticles had on liver and kidney function as there were no changes to the functional indices. As such, the studies utilizing injection and instillation route of exposures need to be validated by appropriately designed inhalation studies utilizing exposures doses relevant to human exposure.

## 8. Overview of Carcinogenicity and Genotoxicity Endpoints

Carcinogenicity refers to the process of cancer formation which can be generally described as two stages encompassing initiation and promotion. Typically, the initiation stage represents heritable genetic changes while promotion occurs when an initiated cell undergoes proliferative and genotypic changes presenting a malignant phenotype. Researchers have studied pathways that are involved with carcinogenesis such as inflammation, hypoxia, oxidative stress, cytotoxicity, and apoptosis through in vitro and in vivo assays.

Mutagenicity refers to the capacity of a substance to form mutations and implies permanent changes in the structure and/or amount of the genetic material of an organism while genotoxicity is a broader term that refers to the capability of a substance to damage DNA and/or chromosomes. NiO nanoparticles have been studied in several in vivo (Table 4) and in vitro (Supplemental Table S2) genotoxicity studies as described in more detail below.

Epidemiology studies involving inhalation exposure to micron-size (inhalable aerosol fraction) particles have provided evidence on an association between oxidic Ni exposure and increased respiratory cancer risk [8,93]. However, there is a lack of epidemiological studies regarding oxidic nickel nanoparticles and associated toxicity effects. Thus, animal studies are relied on to understand the potential carcinogenic effects. Overall, there is limited evidence from in vivo studies that oxidic nickel nanoparticles are carcinogenic. The existing studies described below have limitations and it is necessary to conduct more research using studies that have more realistic dosing (route, method, duration, etc.), appropriate animal models, sufficient animal numbers, extended follow-up time, and appropriate controls (e.g., other particles of different sizes and dosed at different masses). Although chronic inflammation can lead to a tumor-promoting environment, no animal study has evaluated the inflammatory response beyond 12 months post exposure to oxidic nickel nanoparticles. Additionally, pathways that regulate inflammation can be considered tumor promoting rather than tumor initiating. Further, inhalation studies of micron-size nickel compounds have shown that inflammation has been found to be necessary, but not sufficient, to induce tumors in rats [8]. Compounds that initiate tumors do so through direct or indirect mutagenic mechanisms (often confused with the broader term genotoxicity). Pathways such as inflammation, hypoxia, oxidative stress, cytotoxicity, and apoptosis have been studied in vitro by various researchers without consideration of dose relevant to human exposures (Supplemental Table S2). NiO nanoparticles are capable of generating oxidative stress and are cytotoxic to various cell lines. However, the effects are observed at high doses that are not representative of doses expected from human exposure and evidence is lacking suggesting that these effects are related to preneoplastic events.

Sutunkova et al. [72] evaluated the DNA fragmentation in nucleated blood cells in rats exposed to NiO nanoparticles at a concentration of 0.23 mg/m^3^ via nose-only inhalation for four hours per day, five days per week, for 10 months. The authors reported that NiO nanoparticle exposure led to a statistically significant increase in the genomic DNA fragmentation coefficient in circulating nucleated blood cells compared to control animals [72]. Further, the authors noted that oral administration of a bioprotective complex attenuated the DNA fragmentation coefficient [72]. However, the authors reported that NiO nanoparticles induced systemic toxicity at this exposure which could have confounded the results.

Dumala et al. [34] and Saquib [69] studied various genotoxicity endpoints after oral gavage of NiO nanoparticles to rats. Dumala et al. [34] reported dose-dependent, significant increase in % tail DNA in peripheral blood leukocytes, and liver, and kidney cells at 24 h after exposure to 500 mg/kg, a dose-dependent increase in polychromatic erythrocytes micronuclei in bone marrow cells at 24 h after exposure to 250 and 500 mg/kg, and dose-dependent chromosomal aberrations in bone marrow cells at 24 and 48 h after exposure to 250 and 500 mg/kg. Saquib et al. [69] administered NiO nanoparticles via oral gavage at doses 1 to 4 mg/kg/day for 7 or 14 days. Significant increases in chromosomal aberrations were observed for 2 mg/kg/day after a 14 day exposure and for 4 mg/kg/day after 7 and 14 days of exposures, a significant increase in polychromatic erythrocytes and micronucleated erythrocytes after exposure to 4 mg/kg/day for 7 and 14 days, and an increase in % tail DNA after exposure to 4 mg/kg/day for 7 days and 1 to 4 mg/kg/day for 14 days; trends were dose dependent. 

In summary, NiO nanoparticles were reported to induce DNA fragmentation, increase micronuclei frequency in bone marrow cells, and increase chromosome aberrations in bone marrow cells. Interestingly, a single dose of 125 mg/kg NiO nanoparticles did not induce genotoxicity in one study [34], but a daily dose of 2 mg/kg/day for 14 days and 4 mg/kg/day for 7 and 14 days led to genotoxicity in the other study [69]. DNA damage and repair endpoints are considered “indicator tests” by OECD since they do not measure stable genetic damage (OECD 2015). Further, the positive results were observed at either high doses or in animals experiencing systemic toxicity. Thus, future research, i.e., studies that use relevant doses, endpoints, and methodology, is still necessary to understand the genotoxic response associated with NiO nanoparticles.

Regarding in vitro genotoxicity studies, NiO nanoparticles were shown to induce increases in micronuclei frequency, chromosome-type aberrations, DNA strand breaks, and DNA damage. However, most studies utilized methodologies that did not adhere to OECD guidelines (did not score enough metaphases, testing cytotoxic concentrations) and thus care needs to be taken when interpreting these results. Di Bucchianico et al. [32], for example, reported that NiO nanoparticles increased the frequency of micronuclei, nucleoplasmic bridges, nuclear buds, and chromosome-type aberrations in BEAS-2B cells exposed to NiO nanoparticles. Dumala et al. [37] reported that there was a statistically significant, dose-dependent increase in the incidence of micronucleus frequency for lymphocytes dosed with 25 and 50 µg/mL. The authors noted that the cytotoxicity data was correlated with the genotoxicity data and supported the genotoxic abilities of NiO nanoparticles.

Additionally, Akerlund et al. [26] tested the mutagenic potential of NiO nanoparticles by examining gene mutations at the *Hprt* gene locus in mES and V79-4 cells. The authors reported a statistically significant increase in mutation frequency for mES cells exposed to 0.5 µg/mL NiO nanoparticles; however, the effect was not dose-dependent, with no increase in mutation frequency for 1 or 5 µg/mL NiO nanoparticles.

The DNA damage induced by NiO nanoparticles were studied in lymphocytes, BEAS-2B, A549, and human bronchial epithelial cells (HBEC) with exposure to 1, 5, 10 µg/mL NiO nanoparticles resulted in DNA strand breaks in BEAS-2B cells [32]. Åkerlund et al. [26] reported a statistically significant increase in DNA strand breaks, determined via the comet assay, for human bronchial epithelial cells (HBEC) exposed to 5, 10, 25 µg/mL NiO nanoparticles. However, there was no increase in DNA double strand breaks, assessed via γ-H2AX staining, after exposure to NiO nanoparticles. Further, Dumala et al. [37] reported that there was a statistically significant, dose-dependent increase in % of tail DNA and increased incidence of micronucleus frequency for lymphocytes dosed with 25 and 50 µg/mL. The authors noted that the cytotoxicity data was correlated with the genotoxicity data and supported the genotoxic abilities of NiO nanoparticles. However, as described above, the increased cytotoxicity may confound these results, rather than support them [94,95]. Latvala et al. [21] examined DNA damage in A549 cells and reported that NiO nanoparticles induced DNA damage. However, the authors did not examine repair mechanisms or look at recovery of cells after particle exposure. Capasso et al. [30] found that DNA repair pathways were activated after exposure of BEAS-2B cells and A549 cells to NiO nanoparticles. However, all of these endpoints have broad overlapping pathways with many different health outcomes besides cancer, so not necessarily indicative of carcinogenicity.

As noted above, DNA damage and repair endpoints do not measure stable genetic damage and are considered “indicator tests” by OECD [96]. Thus, while genotoxic responses were reported after exposure to NiO nanoparticles in in vitro cells, further research needs to be conducted with methodologies utilizing OECD guidelines to confirm these positive results. Further, there is no evidence that the NiO nanoparticles induced carcinogenic responses.

## 9. Potential Toxic Mechanisms and Comparison to Micron Data

In general, it appears that the bioavailability of nickel at intracellular target areas leads to inflammatory processes that are responsible for the toxicological effects of NiO nanoparticles at high exposure levels. This likely involves the formation of excessive ROS, which can damage various cell structures including lipids, membranes, proteins, and nucleic acids, leading to cytotoxicity and apoptosis [19,21,29]. However, it is not clear if oxidative stress occurs at exposure levels relevant to humans, or whether this stress at the cellular level translates into significant effects at the organ or system level [8]. Both micron- and nano-sized oxidic nickel particles are predicted to follow a similar mode of action with an anticipated difference in potency depending on differential cellular uptake and/or particle dissolution [97,98]. Overall evaluations and comparisons of the evidence presented above appear to support this hypothesis, as the observed toxicity profiles of both oxidic nickel micron-sized particles and nanoparticles do not appear different and new unexpected nano-specific effects have not been observed for oxidic nickel nanoparticles.

Several studies reported greater toxicity (e.g., lung inflammation, organ toxicity, etc.) with NiO nanoparticles compared to the larger NiO particles [19,21,40,65,68,73]. Additional studies suggested NiO nanoparticles could have greater genotoxic/mutagenic potential compared to NiO micron-sized particles [21,34,37,99,100,101]. Although NiO nanoparticles appear to show more toxicity in some studies, this could be due to differences in potency, toxicokinetics, bioavailability, and specific physical/chemical characteristics. Interestingly, none of the in vivo studies reported mortality even at doses several orders of magnitude greater than the European occupational exposure limits proposed for the general nanomaterial category most appropriate for nickel nanoparticles (see Section 5.1).

The small size and large surface area for a given mass or volume of nanoparticles offers enhanced properties compared with larger particles or micron-sized materials of similar composition. Likewise, the increased surface area in relation to mass or volume may also render nanomaterials more biologically reactive (e.g., increase particle surface-cell interactions) as compared to larger sized particles. Although many researchers studied the effects of NiO nanoparticles versus larger size NiO particles in both in vitro and in vivo assays [19,21,40,65,73], the studies did not account for differences in surface area when comparing nano- versus micron-sized particles and thus did not consider the specific surface reactivity [102]. Some assays performed in vitro found that NiO nanoparticles were more active than larger particles on a per weight basis [19]. Ogami et al. [65] and Yu et al. [73] also found that NiO nanomaterials were more biologically active compared with micron-sized nickel particles after intratracheal instillation of equivalent mass doses. One hypothesis is that a greater release of nickel ions results from the increased active surface area of NiO nanoparticles compared with larger sized particles. Among other factors, the dissolution depends on the surface area and therefore, the dose response may be better characterized by surface area instead of weight. Some data suggest that nano and micron-sized particles follow a similar dose response when normalized to specific surface area reactivity [90]. Future research will need to consider comparing nano- versus micron-sized nickel particles according to surface area rather than weight. This may provide a better characterization of the dose response curve and possibly help justify differences in potency.

## 10. Conclusions

A total of 60 in vitro and in vivo mammalian toxicity studies were identified and critically evaluated for health effect endpoints and mechanisms associated with toxicological responses of oxidic nickel nanoparticles. The in vitro literature studied the toxicity associated with NiO and Ni(OH)_2_ nanoparticles; cytotoxicity was the most common endpoint while ROS, apoptosis, and markers of inflammation were also examined in multiple studies. In general, the in vitro studies demonstrate that oxidic nickel nanoparticles have the ability to induce cytotoxicity under certain culture conditions and doses. While in vitro assay data can be potentially useful for hazard identification in the context with other types of data and studying aspects of certain molecular pathways, this kind of data remains limited for human health risk assessment. The in vivo literature studied the toxicity associated with NiO and Ni(OH)_2_ nanoparticles; the most common test model studied was intratracheal instillation of rats, followed by whole body inhalation of mice and rats. Lung inflammation was the most common health endpoint evaluated. Other endpoints included ROS generation, carcinogenicity, cardiovascular, and systemic responses. Oxidic nickel nanoparticles have been documented to cause acute inflammation in various rodent assays; however, recovery was observed in the majority of the studies. The chronic nature of systemic inflammation, beyond twelve months, remains unknown. Potential pathways for inflammatory responses include ROS generation, cytotoxicity, and induction of cytokines.

For future studies, whether in vitro or in vivo, a comparative testing approach should be considered to establish a hazard ranking. Ideally, using toxicologically well-characterized positive and negative benchmark materials against which oxidic nickel compounds can be ranked. In addition, future studies should attempt to characterize the chronic effects of oxidic nickel nanoparticles including longer term studies in the local and distant organs. Most important, relevance of doses for establishing exposure–dose–response relationships are essential for an appropriate study design. Determining the actual dose of nanoparticle delivered to the cell has been shown to be a crucial step that should be measured in future studies.

The carcinogenic potential under relevant exposure conditions has not been fully addressed in the current literature. While it could be expected that oxidic nickel nanoparticles share similar hazards as micron-sized oxidic nickel particles with possible differences in potency, it will be critical to evaluate these responses with validation across multiple experimental designs. In addition, a search into the fate of oxidic nickel nanoparticles deposited in the lung and translocated to secondary organs needs to be considered. Specifically, future work can investigate what happens to NiO nanoparticles at a subcellular level when interacting with cell organelles, proteins and fluids, such as using high-resolution analytical scanning transmission electron microscopy coupled with electron energy loss spectroscopy analysis [103].

The literature regarding oxidic nickel nanoparticles has generated some insights into potential biological responses. However, numerous considerations must be taken into account when interpreting the current data with regard to potential human health effects. Extrapolation from in vitro to in vivo responses as well as rodent assays to human health necessitates careful analysis and in vivo validation. Future research is needed to validate the relevance of in vitro assays compared with in vivo activity and in turn the relevance to human exposures. Additionally, future experiments should examine a wide range of doses that establish an NOAEL for both acute and chronic exposure scenarios, including studying doses correlated with expected human exposure to oxidic nickel nanoparticles. While these concentrations will not be fully known until exposure monitoring is conducted, based on current occupational exposure limits, some studies have tested concentrations several magnitudes higher than the proposed occupational limits for CMAR nanomaterials. Further research is also needed to elucidate the contribution of key nanoparticle characteristics including composition, size, agglomeration and aggregation state, surface morphology, production method, and solubility and dissolution rates in vivo. Finally, the exposure–dose–response relationship for a specific nickel nanoparticle may be characterized in part according to its surface area, which contributes to particle dissolution and potential biological responses.

## Figures and Tables

**Table 2:** In vivo literature associated with lung inflammation and damage endpoints after exposure to oxidic nickel nanoparticles. Studies are arranged by nanoparticle type, followed by exposure method.

Study	Klimisch Score	Model	Dosing Regimen (Exposure Method) (Dose Range and Unit) (Duration/Frequency) (Follow-Up Time)	Health Endpoint (Assay)
**NiO Nanoparticles**				
[38]	K3 ^A,B^	Male Wistar rats	Whole body inhalation, 200 µg/m^3^ or 9.2 × 10^4^ particles/cm^3^ 4 w, 5 d/w, 6 h/d, 3 d and 1 m	Disease profile (lung microarray analysis)
[45]	K3 ^A,B^	Male Wistar rats	Whole body inhalation 200 µg/m^3^ 4 w, 5 d/w, 6 h/d 3 d, 1 m, and 3 m	Lung inflammation (BALF pulmonary surfactant components, BALF surface tension, histopathology)
[64] ^A^	K3 ^A,B^	Male Wistar rats	Whole body inhalation, 200 µg/m^3^ or 9.2 × 10^4^ particles/cm^3^ 4 w, 5 d/w, 6 h/d, 3 d, 1 m, and 3 m	Lung inflammation (histopathology)
[66]	K3 ^A,B^	Male Wistar rats	Whole body inhalation, 1.0 × 10^5^ particles/cm^3^ * or 2.8 µg/m^3^, 4 w, unclear d/w, 6 h/d 4 d, 1 m, and 3 m	Lung inflammation (histopathology) Lung clearance (deposited nickel)
[58]	K3 ^A,B^	Male Wistar rats	Whole body inhalation, 1.0 × 10^5^ particles/cm^3^ * or 2.8 µg/m^3^, 4 w, 5 d/w, 6h/d, 4 d, 1 m, and 3 m	Lung inflammation and fibrosis (BALF cell counts, gene expression, histopathology)
[72]	K3 ^A,B^	Female white rats	Nose-only inhalation 1.0 mg/m^3^ 4 h/d, 5 d 1 d 0.23 mg/m^3^ 4 h/d, 5 d/w, 3, 6, 10 m 1 d	Lung inflammation (BALF cell counts, histopathology)
[39]	K2 ^A^	Male F344 rats	Intratracheal instillation 0.2, 1 mg for instillation, One time dose 3 d, 1 m, 3 m, 6 m Whole body inhalation 320, 1650 µg/m^3^ 4 w, 5 d/w, 6 h/d 3 d, 1 m, 3 m, 6 m	Lung ROS (oxidative stress markers, BALF gene expression)
[59]	K2 ^A^	Male Fischer 344 rats	Intratracheal instillation 0.2, 1 mg One time dose 3 d, 1 w, 1 m, 3 m, 6 m Whole body inhalation 0.32, 1.65 mg/m^3^ 4 w, 7 d/w, 6 h/d 3 d, 1 m, and 3 m	Lung inflammation (BALF cell count, BALF chemokines, BALF LDH, histopathology, morphological features of alveolar macrophages)
[28]	K2 ^A^	Female Balb/c mice	Intratracheal instillation 10, 20, 50, 100 µg One time dose 1, 7, 28, 29 d	Lung inflammation (SPECT analysis, CT analysis) Lung damage (BALF protein levels, BALF LDH, histology)
[29]	K3 ^A,B^	Male Sprague Dawley rats	Intratracheal instillation 800 µg or 3300 µg/kg One time dose 3, 7, 28 d	Lung inflammation (BALF cell counts, BALF ALP, protein levels, Histopathology, cytokines) Lung damage (BALF LDH, BALF protein levels)
[12]	K3 ^A,B^	Female Wistar rats	Intratracheal instillation, 150 cm^2^/rat, or 163.5 µg/rat, One time dose 24 h and 4 w	Lung inflammation (BALF cell counts, BALF protein and lipids, histopathology, cytokine profile) Macrophage function (surfactant clearance)
[13]	K2 ^A^	Female Wistar rats	Intratracheal instillation, 50 and 150 cm^2^/rat, or 54.5–163.5 µg/rat, One time dose 24 h and 4 w	Lung inflammation (BALF cell counts, protein, histopathology, cytokine profile)
[14]	K3 ^A,B^	Female Wistar rats	Intratracheal instillation, 150 cm^2^/rat, or 163.5 µg/rat, One time dose 24 h and 4 w	Lung inflammation (BALF cell counts, BALF LDH, protein, cytokine profile)
[41]	K3 ^A,B^	Male Wistar rats	Intratracheal instillation, 200 µg/rat, One time dose 1 h, 24 h, 72 h, and 1 w	Lung damage and ROS (BALF LDH and protein levels, oxidative stress markers)
[42]	K3 ^A,B^	Male Wistar rats	Intratracheal instillation, 200 µg/rat, One time dose 1 h, 4 h, 24 h, 72 h, and 1 w	Lung damage and ROS (BALF LDH levels, oxidative stress markers)
[49]	K2 ^A^	Female Wistar rats	Intratracheal instillation 50, 100, and 200 cm^2^/rat, or 54.5, 109, 218 µg/rat One time dose 1 d, 2 d, 3 d, and 4 d	Lung inflammation (BALF cell counts, BALF total protein, BALF LDH, cytokine levels, levels of anaphylatoxins) Lung clearance (BALF Ni levels)
[53]	K3 ^A,B^	Female Wistar rats	Intratracheal instillation, 250 cm^2^/rat, or 2700 µg/rat, One time dose 24 h	Lung inflammation (BALF LDH and protein levels, BALF cell counts)
[60]	K3 ^A,B^	Male Wistar rats	Intratracheal instillation, 1000 µg/rat (3300 µg/kg) One time dos e 3 d, 1 w, 1 m, 3 m, and 6 m	Lung inflammation (BALF cell counts, chemokine levels, histopathology)
[61]	K2 ^A^	Male Wistar rats	Intratracheal instillation, 100 and 200 µg/rat (330 or 660 µg/kg) One time dose 3 d, 1 w, 1 m, 3 m, and 6 m	Lung inflammation, fibrosis, and allergy (BALF macrophage counts, BALF alkaline phosphatase release, lung and BALF cytokine profile, histopathology)
[62]	K3 ^A,B^	Male Wistar rats	Intratracheal instillation 1000 mg/rat One time dose 3 d, 1 w, 1 m, 3 m, and 6 m	Lung inflammation (BALF cytokine levels, tissue cytokine levels, histopathology)
[63]	K2 ^A^	Male Wistar rats	Intratracheal instillation, 100 and 200 µg/rat (330 or 660 µg/kg) One time dose 3 d, 1 w, 1 m, 3 m, and 6 m	Lung inflammation (BALF cell counts, chemokine levels, histopathology)
[23]	K2 ^A^	Male Wistar rats	Intratracheal instillation, 100 and 200 µg/rat (330 or 660 µg/kg) One time dose 3 d, 1 w, 1 m, 3 m, and 6 m	Lung inflammation (BALF total protein concentration, BALF phospholipid concentration, BALF surface tension)
[65]	K3 ^A,B^	Male Wistar rats	Intratracheal instillation, 2000 µg/rat, One time dose 3 d, 1 w, 1 m, 3 m, and 6 m	Lung inflammation (BALF cell counts, histopathology, collagen deposition)
[67]	K2 ^A^	Male Fisher rats	Intratracheal instillation 0.2, 1 mg One time dose 3 d, 1 w, 1 m, 3 m, 6 m Whole body inhalation 320, 1650 µg/m^3^ 4 w, 5 d/w, 6 h/d 3 d, 1 m, 3 m	Lung inflammation (histopathology) Lung clearance (deposited nickel)
[70]	K3 ^A,B^	Male F344 rats	Intratracheal instillation 2 mg/kg One time dose or 2–4 divided doses 3, 28, 91 d	Lung inflammation (BALF cell count, BALF protein, histopathology) Lung damage (BALF protein levels, BALF LDH)
[20]	K2 ^A^	Male F344/DuCrlCrlj rats	Intratracheal instillation 0.67, 2.0, or 6.0 mg/kg One time dose 3, 28, 91 d	Lung inflammation (histopathology) Lung clearance (organ nickel burden, modeling)
[44]	K3 ^A,B^	Female Wistar rats	Pharyngeal aspiration 90 cm^2^/rat or 98.1 µg/rat One time dose 1, 28 d	Lung inflammation (BALF cell counts, LDH, protein concentration, BALF cytokine profile, BALF phospholipids)
[15]	K2 ^A^	Male C57BL/6J mice	Pharyngeal aspiration 20, 40, 80 µg/mouse One time dose 1, 7 d	Lung inflammation (WLL cell count, WLL LDH, WLL albumin levels)
**Ni(OH)_2_ Nanoparticles**				
[22]	K2 ^A^	Male C57BL/6 mice	Whole body inhalation, Short-term study: 103.2, 565.0, 1204 µg/m^3^ 4 h, one time dose 24 h Long-term study: 124, 124,5, 129.3 µg/m^3^, up to 5 m, 5 d/w, 5 h/d, 24 h	Lung inflammation (BALF cell counts, BALF protein levels, histopathology, cytokine, chemokine RT-PCR)
[46]	K3 ^A,B^	Male ApoE^-/-^ mice	Whole body inhalation, 100 µg/m^3^, 1 w or 5 m, 5 d/w, 5 h/d, 24 h	Lung ROS/inflammation (ROS markers, mitochondrial DNA damage, BALF cell counts/protein, cytokine, chemokine, histopathology)
[47]	K3 ^A,B^	Male C57BL/6 mice	Whole body inhalation, Ni(OH)_2_ 570 and 1222 µg/m^3^,	Lung inflammation and ROS (BALF cell counts, BALF protein levels, QT-PCR for *Ho-1* and *Ccl-2*)

* Particle number was the only dose metric reported. h: hour; d: day; w: week; m: month; BALF: Bronchoalveolar lavage fluid; LDH: Lactate dehydrogenase; ROS: Reactive oxygen species; QRT-PCR: Quantitative Real Time Polymerase Chain Reaction; ALP: alkaline phosphatase; WLL: Whole lung lavage. ^A^ Not a guideline study. ^B^ Tested limited numbers of doses.

**Table 3:** In vivo literature associated with systemic endpoints after exposure to oxidic nickel nanoparticles. Studies are arranged by nanoparticle type, followed by exposure method.

Study	Klimisch Score	Model	Dosing Regimen (Exposure Method) (Dose Range and Unit) (Duration/Frequency) (Follow-Up Time)	Health Endpoint (Assay)
**NiO Nanoparticles**				
[72]	K3 ^A,B^	Female white rats	Nose-only inhalation 1.0 mg/m^3^ 4 h/d, 5 d 1 d 0.23 mg/m^3^ 4 h/d, 5 d/w, 3, 6, 10 m 1 d	Organ damage (histopathology of liver, kidney, brain, various functional and biochemical indices)
[70]	K3 ^A,B^	Male F344 rats	Intratracheal instillation 2 mg/kg One time dose or 2–4 divided doses 3, 28, 91 d	Organ damage (organ weight of liver, kidney, lung, spleen and brain; histopathology of liver, kidney, lungs, spleen, brain, and pulmonary-related lymph nodes) Hematological analysis (cell count, blood biochemistry)
[73]	K2 ^A^	Male Wistar rats	Intratracheal instillation 0.015, 0.06, or 0.24 mg/kg 2 d/w, 6 w	Liver damage (biomarkers of stress, liver weight, histopathology)
[27]	K2 ^A^	Male Wistar rats	Oral gavage 500, 1000 mg/kg One time dose 14 days	Clinical toxicology (food consumption, body weight, organ weight) Organ damage (various functional and biochemical indices, RBC and WBC count)
[34]	K2 ^C,D^	Female Wistar rats	Oral gavage 5, 50, 300, 2000 mg/kg One time dose 14 d	Organ damage (histopathology of brain, heart, liver, spleen and kidneys) Organ clearance (Ni content) Mortality
[35]	K2 ^A^	Female Wistar rats	Oral gavage 125, 250, 500 mg/kg One time dose 24 h	Organ damage (histopathology of liver, kidney, brain, various functional and biochemical indices)
[36]	K2 ^C,D^	Male and female Wistar rats	Oral gavage 50, 100, 200 mg/kg 28 d, 7 d/w 24 h	Clinical toxicology (food consumption, body weight, organ weight) Organ damage (histopathology of liver, kidney, brain, various functional and biochemical indices)
[48]	K3 ^A,E^	Female rats	Intraperitoneal injection 250, 500 µg/rat 6 w, 3 d/w 24 h	Organ damage (histopathology of liver, spleen, kidney, brain, various functional and biochemical indices) Organ clearance (Ni content of liver, spleen, kidney, brain)
[55]	K3 ^A,D,E^	Male rats	Intraperitoneal injection 10, 25, 50 mg/kg 7 d 12 h	Brain damage (Oxidative stress biomarkers including catalase activity, lipid peroxidation by MDA, Glutathione concentration, total antioxidant capacity; histopathology)
[56]	K3 ^A,B,E^	Female rats	Intraperitoneal injection 500 µg/rat 6 w, 3 d/w	Organ damage (histopathology of liver, spleen, kidney, brain, various functional and biochemical indices) Organ clearance (Ni content of liver, spleen, kidney, brain) Genotoxicity (DNA damage)
[40]	K3 ^A,B^	Female C57BL/6N mice	Pharyngeal aspiration 50 µg/mouse One time dose 21 d	Allergic response (OVA-specific immunoglobulin, gene expression)
**Ni(OH)_2_ Nanoparticles**				
[31]	K2 ^A^	Male C57BL/6 mice	Whole body inhalation, 100, 150, 900 µg/m^3^, 1, 3, or 5 d, 5 h/d, 24 h	Vascular function (carotid artery constriction and relaxation)
[46]	K3 ^A,B^	Male ApoE^−/−^ mice	Whole body inhalation, 100 µg/m^3^, 1 w or 5 m, 5 d/w, 5 h/d, 24 h	Cardiovascular ROS/inflammation (ROS markers, mitochondrial DNA damage, BALF cell counts/protein, cytokine, chemokine) Systemic inflammation (liver SAP protein levels, cytokines/chemokines) Atherosclerosis (plaque formation in aorta, QT-PCR)
[50]	K3 ^A,B^	Male C57BL/6 mice	Whole body inhalation 500 µg/m^3^ 5 h 30 m and 12 h	Hematopoietic damage (bone marrow EPC gene expression, EPC count, EPC chemotaxis, tube formation and proliferation, RT-PCR)
[51]	K3 ^A,B^	C57BL/6 mice	Whole body inhalation, ∼1200 μg/m^3^, 2 d, 5 h/d, ∼700 μg/m^3^, 3 d, 5 h/d, ∼100 μg/m^3^, 5 d, 5 h/d, 24 h	Endothelial progenitor cell effects (cell counts, cell function, cellular signaling pathways)
[52]	K3 ^A,B^	C57BL/6 mice	Whole body inhalation, ∼500 μg/m^3^, 5 h, 0.5 and 12 h	Endothelial progenitor cell effects (cell counts, cell function, cellular signaling pathways) Atherosclerosis (cellular signaling pathways)

h: hour; d: day; w: week; m: month; ^A^ Not a guideline study. ^B^ Tested limited numbers of doses. ^C^ Deviated from OECD guidelines. ^D^ Lacked key details. ^E^ Non-physiological route of administration.

**Table 4:** In vivo literature associated with genotoxic endpoints after exposure to oxidic nickel nanoparticles. Studies are arranged by nanoparticle type, followed by exposure method.

Study	Klimisch Score	Model	Dosing Regimen (Exposure Method) (Dose Range and Unit) (Duration/Frequency) (Follow-Up Time)	Health Endpoint (Assay)
**NiO Nanoparticles**				
[72]	K3 ^A,B^	Female white rats	Nose-only inhalation 1.0 mg/m^3^ 4 h/d, 5d 1 d 0.23 mg/m^3^ 4 h/d, 5 d/w, 3, 6, 10 m 1 d	Genotoxicity (random amplification of polymorphic DNA (RAPD) test)
[34]	K2 ^C,D^	Female Wistar rats	Oral gavage 125, 250, 500 mg/kg One time dose 18, 24 h	Genotoxicity (DNA damage, micronucleus test, chromosomal aberration assay)
[69]	K2 ^A^	Male Wistar rats	Oral gavage 1, 2, 4 mg/kg/day 7 or 14 d, 7 d/w Immediately	Genotoxicity (chromosomal aberrations, micronuclei formation, DNA damage) Cytotoxicity (apoptosis, ROS generation, mitochondrial membrane potential, apoptotic proteins)
[56]	K3 ^A B,E^	Female rats	Intraperitoneal injection 500 µg/rat 6 w, 3 d/w	Genotoxicity (DNA damage)

h: hour; d: day; w: week; m: month; ^A^ Not a guideline study. ^B^ Tested limited numbers of doses. ^C^ Deviated from OECD guidelines. ^D^ Lacked key details. ^E^ Non-physiological route of administration.

## Data Availability

Data sharing not applicable.

## Supplementary Materials

The following are available online at https://www.mdpi.com/2079-4991/11/3/642/s1, Table S1: Physicochemical nanoparticle characteristics reported in the studies investigating the toxicity of oxidic nickel nanoparticles, Table S2: In vitro literature associated with various endpoints after exposure to oxidic nickel nanoparticles.
