# Supplementary material for: Review and Evaluation of the Potential Health Effects of Oxidic Nickel Nanoparticles
Source: Nanomaterials (Basel). 2021 Mar 5;11(3):642. doi: 10.3390/nano11030642 (PMC7999720; doi:10.3390/nano11030642)
Supplement: Supplementary file 1 [file nanomaterials-11-00642-s001.pdf]

Supplemental Information

Review and Evaluation of the Potential Health Effects of Oxidic Nickel Nanoparticles

Sharlee L. More [1,*], Michael Kovochich [2], Tara Lyons-Darden [3], Michael Taylor [3], Alexandra M. Schulte [4] and Amy K. Madl [4]

[1] Cardno ChemRisk, 6720 S Macadam Ave Suite 150, Portland 97219, OR, USA
[2] Cardno ChemRisk, 30 North LaSalle St Suite 3910, Chicago 60602, IL, USA; michael.kovochich@cardno.com
[3] NiPERA, 2525 Meridian Parkway, Suite 240, Durham27713, NC, USA; TLyons-Darden@nipera.org (T.L.-D.); mtaylor@nipera.org (M.T.)
[4] Cardno ChemRisk, 65 Enterprise Drive Suite 150, Aliso Viejo 92656 CA, USA; alex.schulte@cardno.com (A.M.S.); amy.madl@cardno.com (A.K.M.)
* Correspondence: Sharlee.More@Cardno.com

Table S1. Physicochemical nanoparticle characteristics reported in the studies investigating the toxicity of oxidic nickel nanoparticles.

Study	Nanoparticle Type	Oxidic Nickel Nanoparticle Characteristics									
		Agglomeration	Composition	Crystallinity	Particle Size	Purity	Shape	Surface Area	Sur. Charge	Sur. Chemistry	Char. in Media
Abudayyak et al. [24]	NiO	X			X	X	X				X
Ada et al. [25]	NiO	X	X	X	X		X	X			
Åkerlund et al. [26]	NiO	X			X	X	X	X			X
Ali [27]	NiO	X		X	X	X	X				X
Bai et al. [28]	NiO	X	X		X	X	X	X	X	X	X
Cao et al. [29]	NiO	X			X	X	X		X		X
Capasso et al. [30]	NiO	X			X	X	X	X			X
Cho et al. [13]	NiO	X			X			X	X		X
Cho et al. [12]	NiO	X			X		X	X	X		X
Cho et al. [14]	NiO	X			X			X	X		X
Cuevas et al. [31]	$Ni(OH)_2$	X	X		X	X	X			X	X
Di Bucchianico et al. [32]	NiO	X			X	X	X	X		X	X
Duan et al. [33]	NiO	X			X	X	X		X		X
Dumala et al. [34]	NiO	X			X	X	X	X	X		
Dumala et al. [35]	NiO	X		X	X	X	X		X		X
Dumala et al. [36]	NiO	X		X	X		X	X	X		X
Dumala et al. [37]	NiO	X	X	X	X	X	X	X	X		X
Fujita et al. [38]	NiO	X			X		X	X			X
Gillespie et al. 2010 [22]	$Ni(OH)_2$	X	X		X	X	X			X	X
Gutierrez et al. [17]	NiO	X			X			X	X		X
Horie et al. [39]	NiO	X	X		X	X	X				X
Horie et al. [40]	NiO				X	X		X			
Horie et al. [41]	NiO				X						X
Horie et al. [42]	NiO				X			X			X
Horie et al. [43]	NiO		X	X	X	X	X	X			

Study	Nanoparticle Type	Oxidic Nickel Nanoparticle Characteristics									
		Agglomeration	Composition	Crystallinity	Particle Size	Purity	Shape	Surface Area	Sur. Charge	Sur. Chemistry	Char. in Media
Horie et al. [19]	NiO<br>(Green)<br>(Black)	X			X	X		X			X
Jeong et al. [44]	NiO	X			X			X	X		X
Kadoya et al. [45]	NiO	X			X		X	X			X
Kang et al. [46]	$Ni(OH)_2$	X	X		X	X	X			X	X
Kang et al. [47]	$Ni(OH)_2$	X	X		X	X	X			X	X
Katsnelson et al. [48]	NiO		X		X	X	X				X
Latvala et al. [21]	NiO	X			X	X	X	X			X
Lee et al. [49]	NiO	X			X		X	X	X		X
Liberda et al. [50]	$Ni(OH)_2$	X	X		X	X	X			X	X
Liberda et al. [51]	$Ni(OH)_2$	X	X		X	X	X			X	X
Liberda [52]	$Ni(OH)_2$	X	X		X	X	X			X	X
Lu et al. [53]	NiO				X			X			
Lu et al. [54]	NiO				X		X				
Marzban et al. [55]	NiO		X	X	X	X	X	X			
Minigalieva et al. [56]	NiO	X	X		X	X	X				X
Minigalieva et al. [57]	NiO		X		X	X	X				
Morimoto et al. [58]	NiO	X			X	X	X	X			X
Morimoto et al. [59]	NiO	X	X		X	X	X				X
Morimoto et al. [60]	NiO	X			X	X		X			X
Morimoto et al. [61]	NiO	X			X	X		X			X
Morimoto et al. [62]	NiO	X			X	X		X			X
Nishi et al. [63]	NiO	X			X	X	X	X			X
Nishi et al. [23]	NiO	X			X	X	X	X			X
Ogami et al. [64]	NiO	X			X	X		X			X
Ogami et al. [65]	NiO	X			X	X	X	X			
Oyabu et al. [66]	NiO	X			X	X	X	X			X
Oyabu et al. [67]	NiO	X		X	X	X	X	X			X
Pietruska et al. [68]	NiO	X	X	X	X	X	X	X	X	X	X

Study	Nanoparticle Type	Oxidic Nickel Nanoparticle Characteristics									
		Agglomeration	Composition	Crystallinity	Particle Size	Purity	Shape	Surface Area	Sur. Charge	Sur. Chemistry	Char. in Media
Sager et al. [15]	NiO	X					X		X		X
Saquib et al. [69]	NiO	X			X		X		X		X
Senoh et al. [70]	NiO	X			X	X	X				X
Shinohara et al. [20]	NiO	X		X	X		X	X			X
Siddiqui et al. [71]	NiO	X		X	X	X	X				X
Sutunkova et al. [72]	NiO		X		X		X				X
Yu et al. [73]	NiO			X	X	X		X			

Table S2. *In vitro* literature associated with various endpoints after exposure to oxidic nickel nanoparticles.

Study	Nanoparticle (NP) Type	Klimisch Score	Cell Type(s) (Organ or Tissue)	Dosing Regimen: Exposure Method Dose Range & Unit Duration/Frequency Follow-up Time	Health Endpoint (Assay)	Key Findings and Results
Cho et al. [14]	NiO	K2<br><br>Not a guideline study	A549 (Lung)	30, 100, 300 cm²/mL or 32.7-327 μg/mL<br>24 h	Cytotoxicity (Trypan blue exclusion assay)<br>Inflammation (Cytokines, transcription factors)	NiO NPs were cytotoxic and released IL-8 at the highest dose of 300 cm²/mL while the aqueous extracts were all negative. NiO NPs did not induce any changes in the transcription factors AP-1 or NF-kB. Zinc oxide and copper oxide NPs displayed cytotoxicity, IL-8 production, and activation of transcription factors at various lower doses ranging between 3 to 30 cm²/mL. Aqueous extracts of zinc but not copper nanoparticles also displayed cellular effects.
Lu et al. [53]	NiO	K2<br><br>Not a guideline study	A549 (Lung)	9.375-300 cm²/mL<br>100-3,240 μg/mL<br>24 h	Free radical generation (EPR)<br>Oxidative potential (DCF)<br>Cytotoxicity (LDH)<br>Hemolysis (Spectrophotometry)	A wide variety of metal oxide nanoparticles were compared for cell free ROS generation. NiO NPs, cobalt oxide NPs, and carbon black NP induced ROS in both cell free systems (EPR and DCF assay); cerium oxide NP induced ROS production by the EPR method only. Cytotoxicity was dose-dependent for NiO NPs, cerium oxide, and carbon black beginning at 150 cm²/mL. ROS generation in vitro did not correlate with inflammation activity in vivo.
Pietruska et al. [68]	NiO	K2<br><br>Not a guideline study	NCI-H460 (Lung)<br>NHBE (Lung)	5-20 μg/cm²<br>or 8-32 μg/mL*<br>2-72 h	Hypoxia (protein markers)<br>Cytotoxicity (cell number)<br>Apoptosis (caspase activation)	NiO NPs were compared with micron-sized metallic nickel particles for nickel bioavailability, release of ions, activation of HIF-1α, cytotoxicity, and apoptosis. Uptake of NiO NPs was associated with the release of ions from 24 to 48 h. 50% of the available nickel was mobilized in the NiO NPs. NiO NPs induced HIF-1α activation but with different kinetics. NiO NPs induced activation of caspase-3 while the micron-sized particles were negative for caspase-3 activation.

Study	Nanoparticle (NP) Type	Klimisch Score	Cell Type(s) (Organ or Tissue)	Dosing Regimen: Exposure Method Dose Range & Unit Duration/Frequency Follow-up Time	Health Endpoint (Assay)	Key Findings and Results
Capasso et al. [30]	NiO	K2<br><br>Not a guideline study	BEAS-2B (Lung)<br>A549 (Lung)	20, 40, 60, 80, 100 μg/mL<br>45 m, 2 h, 24 h	Cytotoxicity (MTT, Alamar Blue)<br>Inflammation (cytokine levels, protein levels)<br>Oxidation Potential (ROS generation)<br>Apoptosis/necrosis (Hoechst 33342/propidium iodide, cell cycle)<br>Genotoxicity (immunocytochemistry)<br>Cell cycle progression (Flow cytometry)	NiO NPs caused a decrease in cytotoxicity in a dose-dependent manner for both cell lines. There was an increase in apoptotic and necrotic cells in both cell lines. ROS generation was increased after exposure to NiO NPs for BEAS-2B cells, but not A549 cells. For both cell lines, IL-6 and IL-8 were elevated; based on the level of phosphorylated NF-κβ, the level of IkB-α, and levels of JNK and p38, the release of IL-6 and IL-8 was dependent on mitogen activated protein kinases cascade through the induction of NF-kB pathway. There was no increase in IL-1β. NiO NPs caused changes to the cell cycle in both cell lines. Immunocytochemistry showed a significant nuclear translocation of phospho-ATM and phospho-ATR. NiO NPs caused a dose-dependent decrease in G1 phase and a corresponding increase in G2/M phase to A549 cells and an increase in G1 phase and subG1 phase and corresponding decrease in G2/M phase for BEAS-2B cells.
Duan et al. [33]	NiO	K2<br><br>Not a guideline study	BEAS-2B (Lung)	1.25-20 μg/cm²<br>or 2-32 μg/mL*<br>24, 48 h	Cytotoxicity (Cell count)<br>Apoptosis (flow cytometry, caspase-3, protein levels, immunoprecipitation, gene expression, cell transfection)	Ni2+ ions were released within the cell. The NiO NPs caused a dose-dependent decrease in cell viability. Apoptotic cells increased in a dose-dependent manner and the level of caspase-3 was increased. SIRT1, an anti-apoptosis protein, was significantly decreased at both the protein and transcript levels; coimmunoprecipitation assays showed the levels of p53 were decreased, yet there were no significant changes in the transcript levels; however, the acetylation of p53 at the lys382 was significantly increased. Additionally, the expression of Bax was increased after NiO NP treatment. By using resveratrol, an activator of SIRT1, during the exposure to the NiO NPs, the number of apoptotic cells was reduced and the gene levels were not as elevated.

Study	Nanoparticle (NP) Type	Klimisch Score	Cell Type(s) (Organ or Tissue)	Dosing Regimen: Exposure Method Dose Range & Unit Duration/Frequency Follow-up Time	Health Endpoint (Assay)	Key Findings and Results
Di Bucchianico et al. [32]	NiO	K2<br><br>Not a guideline study	BEAS-2B (Lung)	1, 5, 10 µg/mL<br>24 h (SEE NOTE)	Cytotoxicity (Annexin V-FITC/PI staining)<br>ROS generation (fluorescent probes)<br>Genotoxicity (cytokinesis--block micronucleus assay, chromosomal aberration, comet assay	NiO NPs induced apoptosis in a dose-dependent manner. NiO NPs increased the frequency of micronucluei, nucleoplasmic bridges, nuclear buds, and chromosome-type aberrations in BEAS-2B cells exposed to NiO NPs. 1, 5, 10 µg/mL NiO NPs induced DNA strand breaks. NiO NPs induced intracellular ROS.
Gutierrez et al. [17]	NiO	K2<br><br>Not a guideline study	A549 (Lung)<br><br>16HBE14o- (Lung)	0.01, 0.1, 0.5, 10, 100 µg/mL<br>4, 24 h	Inflammation (gene expression)<br>Oxidative stress (gene expression)	Compared the effects of in vitro dispersion techniques on the NP physicochemistry and toxicological effects. Determined that the NiO NPs interfered with the ELISA assay. There was high variability in HO-1 expression among the different NiO concentrations and dispersion medias. One dispersion media produced significant elevation in HO-1, while the other did not. The dispersion media differentially affected NP physicochemical properties.
Latvala et al. [21]	NiO	K2<br><br>Not a guideline study	A549 (Lung)	0.1-40 µg/cm^2<br>or 0.16-64 µg/mL*<br>4, 24, 48 h	Cytotoxicity (Alamar Blue, colony forming efficiency)<br>Oxidation Potential (intracellular ROS)<br>DNA damage (alkaline single cell comet assay)<br>NP localization (TEM, Ni concentration)	All NPs showed increased cytotoxicity in the highest doses and an increase in proliferation at low doses when assayed by the colony forming efficiency. NiO NPs caused the generation of ROS and DNA damage; however, no intracellular ROS was detected for any of the particles. All particles were taken up by the cells within 4 hours and remained in the cell after 24 hour post-incubation.

Study	Nanoparticle (NP) Type	Klimisch Score	Cell Type(s) (Organ or Tissue)	Dosing Regimen: Exposure Method Dose Range & Unit Duration/Frequency Follow-up Time	Health Endpoint (Assay)	Key Findings and Results
Lu et al. [54]	NiO	K2<br><br>Not a guideline study	A549 (Lung)	25-200 μg/mL<br>4 h	Cytotoxicity (MTT, LDH)<br>Oxidation Potential (ROS generation)<br>Apoptosis (Flow cytometry by PI) and distribution of Ni, Fe, Cu and Zn in cells.	NiO NPs were cytotoxic, increased the generation of ROS and increased the number of apoptotic cells. The fluorescent intensity of metal in the cells were in the following pattern: Cu>Zn>Fe>Ni, indicating Ni was the least absorbed material. The metals were located in the perinuclear and cytoplasmic areas. Zinc oxide NPs were the most cytotoxic material and UFP caused the highest levels of ROS and apoptotic cells. The manufactured metal oxide NPs were not as cytotoxic as the environmental UFP particles.
Horie et al. [43]	NiO	K2<br><br>Not a guideline study	A549 (Lung)<br><br>HaCaT (Skin)	10-100,000 μg/mL<br>24 h, 7 d	Cytotoxicity (MTT, LDH assay, clonogenic assay)	1 mg/mL and above displayed notable effects on protein absorption and cytotoxicity for all particles while NiO NPs and zinc oxide NPs induced cytotoxicity at lower concentrations of 0.1 and 0.01 mg/mL, respectively. Salt concentration was able to enhance protein absorption. Aqueous extracts of NiO NPs and zinc oxide NPs induced cytotoxicity while the aqueous extracts of other metal oxide NPs were negative. Ion release was detected for both NiO NP and zinc oxide; however, NiO NP ion release was only marginal at 1 mg/mL compared with 100 mg/mL.

Study	Nanoparticle (NP) Type	Klimisch Score	Cell Type(s) (Organ or Tissue)	Dosing Regimen: Exposure Method Dose Range & Unit Duration/Frequency Follow-up Time	Health Endpoint (Assay)	Key Findings and Results
Horie et al. [19]	NiO (Green) (Black)	K2<br><br>Not a guideline study	A549 (Lung)<br><br>HaCaT (Skin)	100-10,000 μg/mL<br>24 h, 7 d	Cytotoxicity (MTT, LDH assay, clonogenic assay)	Ultra-fine NiO NP cytotoxicity was greater than fine-sized nickel particles; doses for NiO NP toxicity were high and ranged from 0.1 to 10 mg/mL. Aqueous extracts of the NiO NPs also induced cytotoxicity. NiO NPs released greater amounts of ions compared with larger particles on a per weight basis. More ion release was observed in cell media compared with water. Two different crystalline phases of NiO NPs (green vs black) were tested and green NiO NPs induced greater cytotoxicity and greater ion release compared with black NiO NPs. Sedimentation and size stabilization factors were used to determine that aggregation state did not affect the cytotoxic response but was dependent on the concentration of nickel. Cytotoxicity-pulsing experiments were used to display that a 6 h wash returned NiCl2 to control levels while NiO NPs did not return to control levels.
Siddiqui et al. [71]	NiO	K2<br><br>Not a guideline study	HEp-2 (Lung)<br><br>MCF-7 (Breast)	1-100 μg/mL<br>24 h	Cytotoxicity (MTT, NRU)<br>ROS (DCF, lipid peroxidation, GSH)<br>Apoptosis (caspase-3, DNA ladder assay)	NiO NPs induced cytotoxicity, ROS, and apoptosis in a dose-dependent manner starting at 2 μg/mL. Exposure to curcumin (antioxidant) reduced the levels of cytotoxicity, ROS, and apoptosis.
Minigalieva et al. [57]	NiO	K2<br><br>Not a guideline study	MRC-5 (Lung)<br>THP-1 (Blood)<br>SH-SY5Y (Bone marrow)	3.125, 6.25, 12.5, 25, 50 μg/mL<br>24 h	Cytotoxicity (MTS, ATP, tyrosine hydroxylase)	NiO NPs were cytotoxic in a dose-dependent manner. Mn_3O_4 NPs were more cytotoxic than NiO NPs. Ni NP dissolution was dependent on the media it was suspended in and the addition of fetal bovine serum enhanced dissolution.

Study	Nanoparticle (NP) Type	Klimisch Score	Cell Type(s) (Organ or Tissue)	Dosing Regimen: Exposure Method Dose Range & Unit Duration/Frequency Follow-up Time	Health Endpoint (Assay)	Key Findings and Results
Åkerlund et al. [26]	NiO	K2<br><br>Not a guideline study	HBEC (Lung)<br>mES (embryonic stem cells)	5, 10, 25, 50 μg/mL<br>24 h	Cytotoxicity (Alamar blue; Histone γ-H2AX)<br>Genotoxicity (Comet assay, Hprt mutation assay, ToxTracker reporter)<br>ROS generation (fluorescent probes)	NiO NPs were not cytotoxic at any tested dose. There was a statistically significant increase in DNA strand breaks for HBECs exposed to 5, 10, 25 μg/mL NiO NPs. However, there was no increase in DNA double strand breaks, assessed via γ-H2AX staining, after exposure to NiO NPs. Additionally, NiO NPs induced ROS generation. Results from the ToxTracker reporter lines suggest that NiO NPs trigger the oxidative stress reporter Srxnl and Ddit3 report (protein stress). There was a statistically significant increase in mutation frequency for mES cells exposed to 0.5 μg/mL NiO NPs. There was no increase in mutation frequency for 1 or 5 μg/mL NiO NPs.
Dumala et al. [37]	NiO	K2<br><br>Not a guideline study	HPBL Lymphocytes (Immune)	0.1, 1, 5, 10, 20, 50 and 100 μg/mL<br>24 h	Cytotoxicity (trypan blue, apoptosis)<br>Genotoxicity (cytokinesis--block micronucleus assay, comet assay)<br>ROS generation (fluorescent probes)<br>Lipid peroxidation	The 50% inhibitory concentration after 24 hours of treatment was estimated as 23.58 μg/mL. There was a statistically significant increase in the incidence of micronucleus frequency for lymphocytes dosed with 25 and 50 μg/mL NiO NPs. Further, there was a statistically significant increase in % of tail DNA and increased incidence of micronucleus frequency for lymphocytes dosed with 25 and 50 μg/mL. The authors noted that the cytotoxicity data was correlated with the genotoxicity data. NiO NP exposure also induced ROS generation and lipid peroxidation.
Abudayyak et al. [24]	NiO	K2<br><br>Not a guideline study	SH-SY5Y (Brain)	0-500 μg/mL<br>24 h	Cytotoxicity (MTT, NRU)<br>Genotoxicity (Comet Assay)<br>Oxidative potentials (enzymes)<br>Apoptotic potentials (Annexin V-FITC)	NiO NPs were taken up by the cells in a dose-dependent manner. 229.34 μg/mL caused a 50% inhibition in cell viability. Additionally, NiO NPs caused dose-dependent DNA damage and apoptosis and significantly induced oxidative damage. TEM images showed that 50 and 150 μg/mL caused ultrastructural changes to the cells, invisible mitochondria, indentations of nuclear membrane, abnormal nuclei with nuclear fragmentation, and chromatin condensation. Additionally, cytoplasmic vacuoles with internalized particles were observed.

Study	Nanoparticle (NP) Type	Klimisch Score	Cell Type(s) (Organ or Tissue)	Dosing Regimen: Exposure Method Dose Range & Unit Duration/Frequency Follow-up Time	Health Endpoint (Assay)	Key Findings and Results
Cao et al. [29]	NiO	K2<br><br>Not a guideline study	Raw 264.7 (Immune)	1, 2, 4 μg/cm^2<br>or 1.6-6.4 μg/mL*<br>12 h<br><br>2 μg/m^2<br>or 3.2 μg/mL*<br>0, 6, 12, 24 h	Cytotoxicity (proliferation)<br>Inflammation (cytokine levels, mRNA levels, protein levels)<br>Oxidation Potential (ROS generation)	NiO NPs induced cytotoxicity in a dose- and time-dependent manner. The Ni^{2+} ion concentration increased in a dose-dependent manner. NiO NPs increased the release of cytokines IL-1β in a dose- and time-dependent manner, but did not affect the levels of TNF-α; the mRNA levels of IL-1β and TNF-α were not increased. Based on cytokine secretion levels, protein levels, and using siRNA and a Nlrp3 knockdown, it was determined that NiO NP mediated IL-1β release was dependent on Nlfrp3/caspase-1 activation. NiO NPs induced ROS generation in a dose-dependent manner. Further, through experiments designed with NAC or cytochalasin D, they showed that NiO NP-induced Nlrp3 inflammasome activation coincidently required phagocytosis and ROS production.
Ada et al. [25]	NiO	K2<br><br>Not a guideline study	HeLa (Cervix)	50-500 μg/mL<br>2-16 h	Cytotoxicity (cell counts)<br>Apoptosis (double and M30 staining)	Cytotoxicity and induction of apoptosis were dose- and timedependent. Cytotoxicity increased at 50 μg/mL NiO NP and higher. Apoptosis pathway was not determined and statistics for individual doses were not clear.

NiO NP: Nickel oxide nanoparticles; MTT: (3-(4,5-dimethylthiazol-2-yl)-2,5-diphenyltetrazolium bromide; LDH: lactate dehydrogenase; NRU: Neutral Red Uptake; GSH: Glutathione; DCF: 2',7'-dichlorofluorescein; TEM: Transmission electron microscopy; PI: Propidium iodide; EPR: Electron Paramagnetic Resonance; ROS: Reactive oxygen species
